# Characteristics of Interventions Targeting Multiple Lifestyle Risk Behaviours in Adult Populations: A Systematic Scoping Review

**DOI:** 10.1371/journal.pone.0117015

**Published:** 2015-01-24

**Authors:** Kristel King, Nick Meader, Kath Wright, Hilary Graham, Christine Power, Mark Petticrew, Martin White, Amanda J. Sowden

**Affiliations:** 1 Centre for Reviews and Dissemination, University of York, York, United Kingdom; 2 Department of Health Sciences, University of York, York, United Kingdom; 3 Population, Policy and Practice, University College London Institute of Child Health, London, United Kingdom; 4 Department of Social and Environmental Health Research, London School of Hygiene and Tropical Medicine, London, United Kingdom; 5 MRC Epidemiology Unit and the Centre for Diet & Activity Research (CEDAR), University of Cambridge, Cambridge, United Kingdom; Örebro University, SWEDEN

## Abstract

**Background:**

Modifiable lifestyle risk behaviours such as smoking, unhealthy diet, physical inactivity and alcohol misuse are the leading causes of major, non-communicable diseases worldwide. It is increasingly being recognised that interventions which target more than one risk behaviour may be an effective and efficient way of improving people’s lifestyles. To date, there has been no attempt to summarise the global evidence base for interventions targeting multiple risk behaviours.

**Objective:**

To identify and map the characteristics of studies evaluating multiple risk behaviour change interventions targeted at adult populations in any country.

**Methods:**

Seven bibliographic databases were searched between January, 1990, and January/ May, 2013. Authors of protocols, conference abstracts, and other relevant articles were contacted. Study characteristics were extracted and inputted into Eppi-Reviewer 4.

**Results:**

In total, 220 studies were included in the scoping review. Most were randomised controlled trials (62%) conducted in the United States (49%), and targeted diet and physical activity (56%) in people from general populations (14%) or subgroups of general populations (45%). Very few studies had been conducted in the Middle East (2%), Africa (0.5%), or South America (0.5%). There was also a scarcity of studies conducted among young adults (1%), or racial and minority ethnic populations (4%) worldwide.

**Conclusions:**

Research is required to investigate the interrelationships of lifestyle risk behaviours in varying cultural contexts around the world. Cross-cultural development and evaluation of multiple risk behaviour change interventions is also needed, particularly in populations of young adults and racial and minority ethnic populations.

## Introduction

Modifiable lifestyle risk behaviours such as smoking, unhealthy diet, physical inactivity and alcohol misuse are the leading causes of major, non-communicable diseases worldwide [1]. In 2008, 36 million deaths (63% of all deaths globally) were linked to cardiovascular diseases, chronic respiratory diseases, cancers, and diabetes.

Large nationally representative surveys have shown that adults often engage in two or more of these risk behaviours at any one time. Prevalence rates of multiple risk behaviours in adult populations have been reported as 68% in England [2], 55% in the Netherlands [3], 52% in the United States (US) [4], and 59% in Brazil [5]. There is also evidence to suggest that risk behaviours can cluster within individuals [2, 6–8]. Clustering has been defined as co-occurring risk behaviours, greater than would be predicted by probability rules, or as underlying patterns in risk behaviours that are identified through more advanced statistical methods [9].

The adoption and maintenance of healthy behaviours, like regular physical activity and eating a healthy diet, are key to preventing chronic, non-communicable disease [10]. Interventions to change risk behaviours have tended to focus on a single behaviour [11, 12] but it is increasingly being recognised that interventions which target more than one risk behaviour may be a more efficient way of improving people’s lifestyles [13]. Such interventions have the potential for greater health benefits, maximisation of health promotion opportunities, more adequate tailoring to participants’ behavioural profiles, and reduction of health care costs [14].

Targeting of multiple risk behaviours is being incorporated into government public health strategies around the world, including the US [15], the Netherlands [16], Australia [17], Sweden [18], and the United Kingdom (UK) [19]. Yet, despite increased interest, little is known about the nature and size of the global evidence base for these interventions. To gain a better understanding of the extent, range and nature of interventions targeting multiple risk behaviours, we undertook a systematic scoping review. Scoping reviews are useful for summarising a collection of evidence with regards to the breadth and depth of a field [20]. Their inclusive approach provides a mechanism to map the existing literature; they are therefore particularly relevant to emerging research fields, such as that of multiple risk behaviours.

We aimed to identify and map all studies that had evaluated the effectiveness of multiple risk behaviour change interventions delivered to adults (aged 16 years or over). This only included adults drawn from general populations or populations at risk of major, non-communicable disease. By performing this mapping exercise, we were also able to identify gaps in this research area.

## Methods

**Figure 1:**
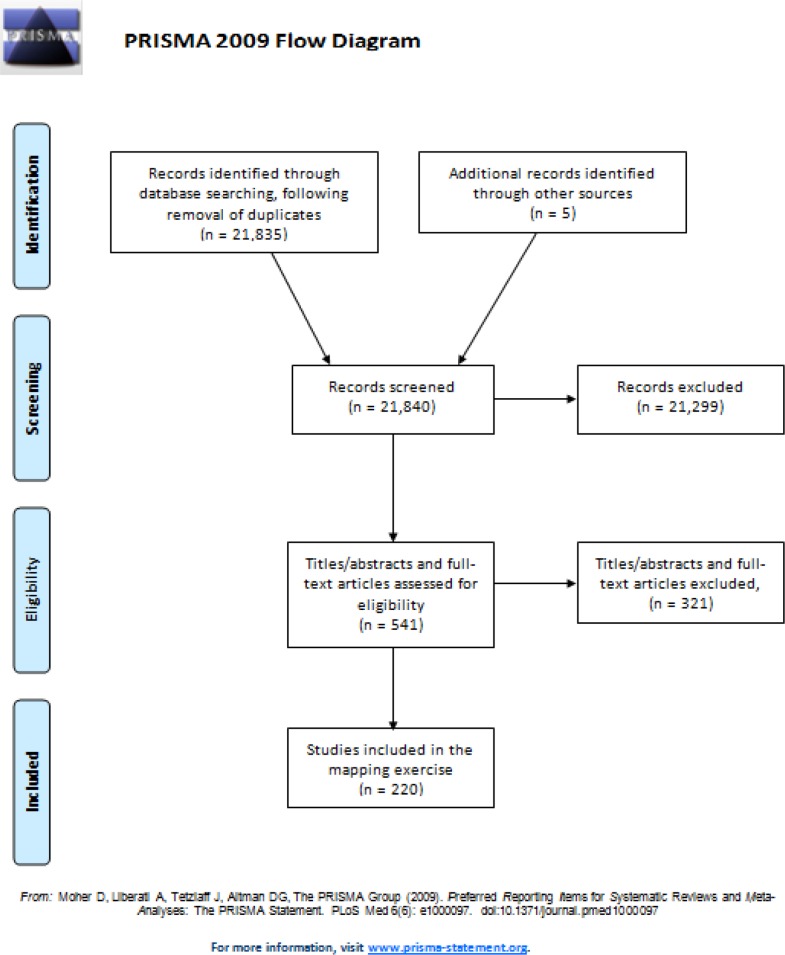
Flow diagram of the study selection process.

The PRISMA (Preferred Reporting Items for Systematic Reviews and Meta-Analyses) checklist and flow diagram were used as aids in the reporting of this scoping review (presented in S1 File and Fig. 1 respectively).

We searched six electronic bibliographic databases (MEDLINE, EMBASE, PsycINFO, Science Citation Index, Cochrane Central Register for Controlled Trials, Applied Social Sciences and Index and Abstracts) for papers published between January, 1990, and January, 2013. We restricted the start date to 1990 because previous scoping searches of the literature have shown that most relevant studies were conducted after 1990. PubMED was also searched from January, 2012 to May, 2013 to ensure that recently published studies were identified. PubMED is one of the most up-to-date databases and includes “ahead of print” and “in process citations” [21]. Searches were not restricted by language or study design. The full search strategy is provided in S2 File.

Inclusion/exclusion criteria were as follows:

***Population*:** Participants aged 16 years or over, drawn from the general population and/or an at-risk population. At-risk populations had biological or genetic risk factors for major, non-communicable diseases such as cardiovascular disease, cancers, and diabetes. Examples of risk factors include medical conditions (e.g., hypertension, metabolic syndrome), previous diagnoses of major diseases (e.g., survivors of cancer) and family history of major disease (e.g., siblings of patients with cardiovascular diseases). Studies including participants aged 15 years or under were excluded to avoid duplication with another, ongoing systematic review [22].

***Intervention*:** Non-pharmacological (or predominantly non-pharmacological) interventions targeting change in at least two risk behaviours (e.g., smoking and physical inactivity, or smoking, physical activity and alcohol misuse). No specific restrictions were applied in relation to the ‘components’ of the intervention, i.e., the behaviour change techniques employed and procedures for their delivery [23]. Procedures for delivery, in this instance, include the people who deliver or receive the intervention, the frequency of sessions, the intervention duration, and the format and context of the intervention. To avoid duplication with an ongoing systematic review [22], studies of school- or family-based interventions were excluded.

***Outcomes*:** Outcomes which demonstrated measurement of the targeted risk behaviours at intervention endpoint/follow-up.

***Study design*:** Any study design which attempted to evaluate the effectiveness of an intervention was eligible for inclusion.

Titles and abstracts of records identified by the searches of the electronic databases were downloaded into Endnote and screened by one reviewer to exclude any obviously irrelevant studies. Remaining titles and abstracts were fully screened for inclusion by two independent reviewers. Where further information was required for screening and/or the mapping exercise, study papers were ordered and assessed independently by two reviewers. Any discrepancies were resolved by consensus, involving a third reviewer where necessary.

Authors of protocols, conference abstracts, and articles describing studies whose results had not yet been reported were contacted to obtain further information. One reviewer extracted descriptive characteristics using Eppi-Reviewer 4 software. Study characteristics were mapped according to study design, country, population characteristics (e.g., ethnicity, gender, age), risk behaviours targeted, sample size, intervention components, and outcomes (including outcome measures) reported. Where papers were reported in languages other than English [24–26], we were able to extract most study characteristics from the English language abstracts.

## Results

A total of 21,835 records were identified through the searches of electronic databases. Fourteen authors of protocols, conference abstracts and study articles without published results were contacted. Nine authors replied; three stated that the full results had not yet been published and six provided the references for published results. After screening titles/abstracts and study papers (where necessary) of all potentially relevant studies identified (541 studies), 220 studies (including five found through contact with authors) met the inclusion criteria and were mapped (Fig. 1). The results from this mapping exercise are presented in S1–S4 Tables.

Most of the studies were randomised controlled trials (RCTs) (62%) and sample sizes ranged from 12 to 28,000. Just under half of the studies were conducted in the US (49%). Participants were most frequently recruited from general populations (14%), or subgroups of general populations (45%) (e.g., university students, racial and ethnic minority groups, older adults). The most commonly targeted risk behaviour combination was diet and physical activity (55%). The focus of most interventions was prevention or reduction of risk for chronic disease (49%), or health promotion (27%). Most interventions had multiple components, including education, advice, counselling, skills training, and incentives. Interventions were often delivered in healthcare practices or clinics, fitness centres, community centres, or university campuses. Behaviour change outcomes varied across the studies, particularly in relation to diet. Other outcomes included changes in serum biomarkers, anthropometric variables, weight, quality of life, knowledge, attitudes, and self-efficacy.

### Year of publication

**Figure 2:**
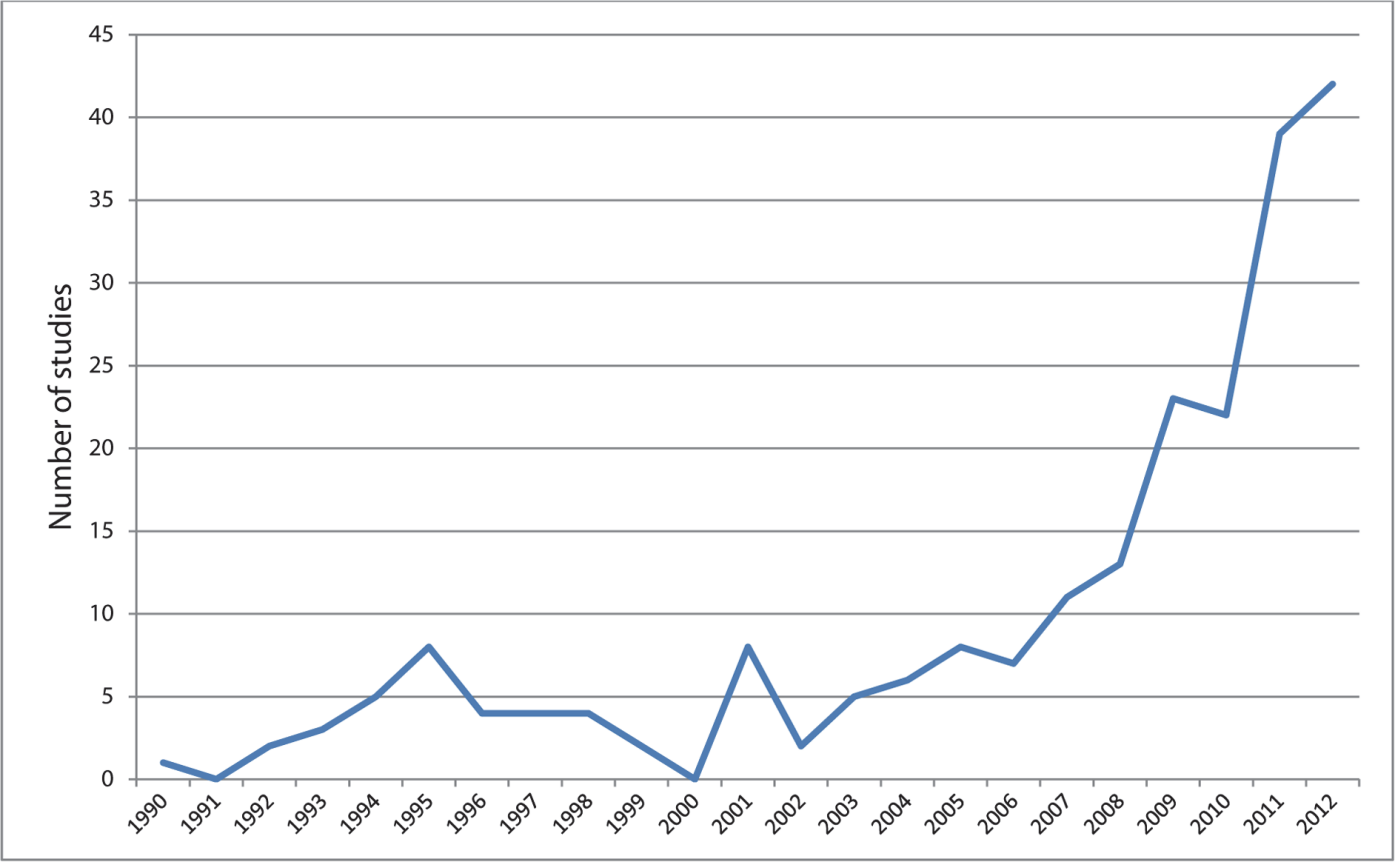
Line graph showing the number of studies published per year since 1990.

Fig. 2 shows the publication rate by year, up to 2012. It demonstrates a steady increase from 2002 onwards, and a sharp increase from 2010 onwards.

Among the 106 studies published between 2010 and January 2013, most were conducted in the US (46 studies), followed by Australia (13 studies), and the Netherlands (10 studies). During the same time period only four UK based studies were published. One study was performed in multiple countries: the UK, the US, and Canada [27].

### Geographical location

**Figure 3:**
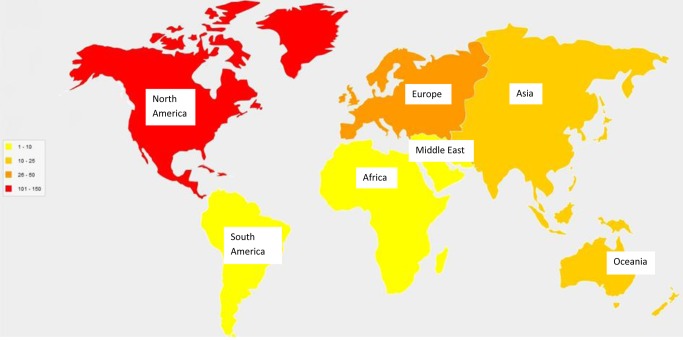
Diagram showing the number of studies conducted in each continent since 1990.

Fig. 3 provides a broad overview of the number of studies that have evaluated multiple risk behaviour interventions in each continent. Most studies were conducted in North America (113 studies), followed by Europe (48 studies), Oceania (21 studies), Asia (17 studies), the Middle East (four studies), Africa (one study), and South America (one study). One study was conducted in North America and Europe [27], and a further 14 studies did not report the country where the study had been conducted. Among these, the authors of the studies were based in the US (eight studies), the Netherlands (one study), the UK (one study), Canada (one study), Japan (one study), Italy (one study), and Germany (one study). All four studies from the Middle East were conducted in separate countries. Other countries that had published single studies on multiple risk behaviour interventions include the following: Mauritius, Vietnam, Taiwan, Mexico, Chile, Poland, Romania, Norway, France, Russia and Northern Ireland. Two of these (Mauritius in Africa and Chile in South America) were the only countries within entire continents to perform such studies.

### Populations

Overall, 30 studies included general adult populations. An additional 100 studies included subgroups of general adult populations, which we classified as ‘targeted’ or ‘non-targeted.’ We defined targeted subgroups as those that had undergone a form of screening (e.g., anthropometric measurements) to confirm eligibility prior to enrolment in the study. This included overweight/obese people (33 studies), adult drug users (11 studies), and university students with problematic substance use (two studies). We defined non-targeted subgroups as those that had not taken part in any screening process. This included university students (15 studies), racial and minority ethnic groups (eight studies), older adults (six studies), and the homeless, or people with low socio-economic status (six studies). Some studies included populations with biological or genetic risk factors for major non-communicable diseases (54 studies). A subset of these studies specified that participants were at risk of particular diseases such as cardiovascular disease (11 studies), or diabetes (eight studies).

Across all of the studies, the UK was the only country that evaluated the use of multiple risk behaviour interventions among young adults other than students (aged 16–25 years) (two studies). Similarly, the US was the only country that focused on racial and minority ethnic groups (eight studies).

### Risk behaviours targeted

Across the 220 studies, a range of behaviours were targeted. These included: dietary behaviours (186 studies), physical activity (181 studies), smoking (64 studies), alcohol use (38 studies), illicit drug use (22 studies), sexual risk behaviours (15 studies), sedentary behaviours (nine studies), seat belt use (four studies), and sun protection /sunbathing (four studies). The most commonly targeted behaviour combinations were diet and physical activity (123 studies), followed by smoking, diet and physical activity (22 studies), smoking, alcohol, diet and physical activity (17 studies), and illicit drug use and sexual risk behaviours (10 studies).

### Interventions

The focus of the interventions included prevention or reduction of risk for chronic disease (107 studies), general health promotion (59 studies), weight management (49 studies), reduction of risk for substance dependence and other problems (three studies), and prevention of initiation of risk behaviours (two studies). Interventions were often multi-component in nature. Common components included health education, advice (sometimes tailored to the individual), lifestyle counselling/coaching, and skills training (including behavioural strategies like goal setting, self-monitoring, etc). Where reported [28, 29], structural changes related to availability and accessibility of resources (e.g., healthy food fairs, physical activity programs), physical structures (e.g., provision of exercise equipment in churches), social structures (e.g., allowing physical activity breaks during long meetings), and cultural and media messages (e.g., through church channels including bulletin inserts, flyers and announcements). Some interventions included cognitive behavioural therapy or motivational interviewing to facilitate behaviour change.

Interventions were delivered in a variety of settings, including healthcare practices/clinics, workplaces, fitness centres, community centres, and university campuses.

Interventions were delivered at the individual level (93 studies), the group level (31 studies), the community level (11 studies), or the population level (three studies). Additional studies reported delivery of interventions at multiple levels (55 studies). Twenty-seven studies did not indicate the level at which their intervention had been delivered.

Interventions delivered face-to-face (where reported) were delivered by university students, lay people, community leaders, researchers, or health professionals (e.g., dieticians, nutritionists, nurses, exercise instructors, GPs, health or exercise psychologists, counsellors, physiotherapists). Where interventions were not delivered face-to-face, they were often delivered by computer and other electronic media (e.g., videos), telephone, and printed materials.

### Study design

Most studies were RCTs (136 studies). Furthermore, the RCT was the most common study design in the US (69 trials), Australia (12 trials), the Netherlands (12 trials), the UK (seven trials), Japan (four trials), Belgium (three trials), Denmark (two trials), Spain (two trials) and Italy (two trials). Where only singular studies had been performed in countries (15 in total), the RCT was the chosen design in five countries: Mauritius, Mexico, Jordan, Romania, and Norway. An additional study carried out in the US, Canada and the UK was also an RCT [27].

Among all remaining studies, designs included non-randomised controlled trials (23 studies), before and after studies (55 studies), case control studies (two studies), one cohort study, one interrupted time series, and one time series repeated measures design. One of these studies did not report its study design.

## Discussion

The objective of this scoping review was to summarise (identify and map) the global evidence base for interventions targeting multiple risk behaviours in general adult populations, or adult populations at risk of major, non-communicable diseases. As very little was known about the composition and size of the evidence base the findings were used to inform a systematic review evaluating the effectiveness of these interventions.

To our knowledge, this scoping review presents the most comprehensive and up-to-date picture of multiple risk behaviour interventions in these populations. Two hundred and twenty studies met our inclusion criteria, most of which were RCTs. Nearly half of the studies were conducted in the US, and most others were undertaken in Europe, Oceania or other countries in North America. The combination of behaviours most commonly targeted was diet and physical activity. Interventions tended to be multi-component in nature (e.g., education, advice, counselling, skills training) and were delivered in a variety of settings, including healthcare practices/clinics, workplaces, fitness centres, community centres, and university campuses.

Most countries, including the US, Canada, Australia, New Zealand, Japan, the UK, Belgium, France, Chile, Norway and Mexico, targeted interventions at both diet and physical inactivity. The focus on this particular combination of behaviours is likely due to the fact that they (i.e., energy intake and expenditure) are major drivers of the growing global obesity epidemic [30]. The considerable interest in these behaviours might also be, in part, linked to the introduction of the World Health Organization’s (WHO) global strategy on diet, physical activity and health in 2004 [31], the overall goal of which was to guide sustainable actions to reduce rates of mortality and morbidity associated with these two risk behaviours. In particular, one of the recommendations stated that governments should promote applied research, with examples including evaluations of the key determinants of effective intervention programmes for diet and/or physical inactivity. Around the same time period, an increased interest in multiple risk behaviour change research seemed to occur. For example, in 2002, the Society of Behavioral Medicine in the US formed a special interest group, focusing specifically on the inter-relationships between health behaviours, and on interventions designed to promote change in multiple health behaviours [32].

Our findings also show that from 2010 onwards, there was a sharp increase in the number of published studies (106 in total) evaluating the effectiveness of multiple risk behaviour change interventions in adults. At around the same time, the countries from which most of the studies originated including the US, the Netherlands and Australia, had either recently or were in the process of prioritising lifestyle risk behaviour change in their national public health strategies [15–17]. A few years earlier the WHO had also published an action plan for the prevention and control of non-communicable diseases [33], with specific mention of four main lifestyle risk behaviours: smoking, alcohol misuse, poor diet, physical inactivity. It is likely that the increased emphasis on these risk behaviours in public health policy, both globally and nationally, is a key factor driving this sudden rise in studies.

Several research gaps were identified through this scoping review. For example, the continents of Africa and South America each had only one study evaluating a multiple risk behaviour change intervention in adults. These are in stark contrast to other continents like North America, and Europe, which were found to have 113 and 48 studies respectively. This possibly reflects differences in research funding and priorities between developed and developing countries. Rates of mortality from infectious, communicable diseases are substantially higher in developing countries [34]. As a result, the global health community has tended to focus on the control of infectious diseases (e.g., HIV/AIDS) within low and middle-income countries, although non-communicable diseases are increasing rapidly and are now more prevalent than infectious diseases in these countries [35]. This increase in the burden of non-communicable disease, and the known relationship between lifestyle risk behaviours and subsequent morbidity, suggest that multiple risk behaviour research is urgently needed in these populations. Specifically, we need to know which interventions are effective in reducing which combination of risk behaviours and understand the mechanisms and contexts that facilitate change. If interventions were shown to be effective in preventing or reducing multiple risk behaviours, they could help to prevent an escalation of chronic health problems within low and middle-income countries in future years.

Other identified gaps include a lack of research focusing on particular population groups, such as young adults other than students (aged 16–25 years), and racial and minority ethnic groups. The UK was the only country that had performed studies evaluating the use of multiple risk behaviour change interventions in young adults. Similarly, the US was the only country to evaluate interventions among racial and minority ethnic groups. This is surprising, given the increase in prevalence of risk behaviours (e.g., low physical activity, high salt intake) in some of these groups in the UK [36], and very little research appears to have been done elsewhere in the world. Perhaps even more surprising is the lack of studies focusing on young adults because many risk behaviours are established during adolescence and carried on into adulthood [37]. Evidence also exists for co-occurrence or clustering of multiple risk behaviours in young adults outside of the UK, for instance in Germany [38], the US [39, 40], Australia [41], and Canada [42, 43]. Evidence about the effectiveness of interventions to prevent or reduce multiple risk behaviours among young adults in these other countries is needed, as is evidence relating to racial and minority ethnic groups globally.

In another review, we previously examined the clustering and co-occurrence of risk behaviours among adults in the UK, and found evidence of clustering between alcohol misuse and smoking, and co-occurrence between unhealthy diet and smoking. The data for these analyses were all consistent and came from large-scale surveys conducted in Scotland (N = 6,574)[44], England (N = 11,492, N = 11,214)[2, 45], and Wales (N = 7,979)[46]. Clustering of alcohol misuse and smoking has also been demonstrated among adults (N = 16,789) in the Netherlands [3]. Among the 220 interventions mapped in this scoping review, none targeted alcohol misuse and smoking, and only seven targeted unhealthy diet and smoking. This highlights a potential disparity between evidence regarding the interrelationships between multiple risk behaviours, and the combinations of behaviours targeted by multiple risk behaviour interventions. When planning future interventions, these initial findings should be considered in order to maximise potential effectiveness in target populations.

Given the broad inclusion criteria of this scoping review, there were many differences (i.e., heterogeneity) between the studies in terms of population, risk behaviour combinations targeted, country, study design and types of intervention (see S1–S4 Tables). Future reviewers evaluating the effectiveness of these interventions should consider using a standardised tool to extract implementation data (e.g., the Oxford Implementation Index [47]), and explore any heterogeneity between studies in their analyses. Intervention characteristics are one potential source of heterogeneity; these include methods of delivery, intervention duration, characteristics of individuals delivering the interventions, and intervention function/s (which can be classified using the Behaviour Change Wheel [48]). Contextual factors are another source of heterogeneity and include population characteristics, the study setting, the publication period of the study, and follow-up duration.

This scoping review has several strengths, including the breadth of the searches undertaken. We searched seven major databases and efforts were made to contact authors of protocols, conference abstracts, and articles describing studies the results of which had not yet been reported. Furthermore, no restrictions were made on language or study design to reduce the risk of relevant studies being missed. The study selection process was performed in duplicate, reducing the risk of reviewer error or bias. An additional strength included the deliberate use of broad inclusion criteria, which enabled us to map most of the evidence base relating to adult populations.

A limitation of this review is inherent in the fact that its purpose was to scope and map the literature without describing the studies in detail. Nevertheless, the objective of this review was achieved because it has increased our knowledge and understanding of the global evidence base, informed an on-going systematic review evaluating the effectiveness of multiple risk behaviour interventions, and highlighted gaps in the intervention literature.

## Conclusions

In conclusion, most of the studies identified and described in this scoping review are randomised controlled trials of interventions aiming to change diet and physical activity in general adult populations, or subgroups of general populations residing in the US. High-quality research is required to investigate the interrelationships of lifestyle risk behaviours in varying cultural contexts worldwide. Cross-cultural development and evaluation of multiple risk behaviour change interventions is also needed, particularly in populations of young adults and racial and minority ethnic populations.

## Supporting Information

S1 File Completed PRISMA checklist. (DOCX)

S2 File Search strategy. (DOCX)

S1 Table Study characteristics (presented according to population). (DOCX)

S2 Table Intervention characteristics (presented according to population). (DOCX)

S3 Table Study characteristics (presented according to study country). (DOCX)

S4 Table Intervention characteristics (presented according to study country). (DOCX)
