# Supplementary material for: Characteristics of Interventions Targeting Multiple Lifestyle Risk Behaviours in Adult Populations: A Systematic Scoping Review
Source: PLoS One. 2015 Jan 24;10(1):e0117015. doi: 10.1371/journal.pone.0117015 (PMC4305300; doi:10.1371/journal.pone.0117015)
Supplement: S2 Table — (DOCX) [file pone.0117015.s004.docx]

**Table S2.** Intervention characteristics (presented according to population)

| **STUDY POPULATION** | **FOCUS OF THE INTERVENTION** | **TOTAL NUMBER OF STUDIES** | **DESIGNS AND COUNTRIES OF STUDIES**  **(NUMBER OF STUDIES PER COUNTRY AND THE TYPE OF INTERVENTION)** |
| --- | --- | --- | --- |
| **GENERAL POPULATION (83 STUDIES, WITH SAMPLE SIZES RANGING FROM 12 TO 5,160):GERAL** ERAL POPULATION (8STU | | | |
| General adult population (aged ≥ 16 years)  (30 studies) | Health promotion | 6 | **RCTs***: US (1-education and counselling with peer), UK (1-CBT** and nicotine replacement therapy)  **Other designs***:** New Zealand (1-education, community activities and structural changes), US (1-coaching and home visits), Sweden (1-education and health examinations), Vietnam (1-advertising) |
|  | Weight management | 5 | **RCTs**: US (2-education and advice, CBT and goal setting)  **Other designs:** Canada (1-education and CBT), country not reported (1- smartphone application), US (1-prescribed diet and exercise activities) |
|  | Prevention/reduction of risk for chronic disease | 19 | **RCTs**: Mauritius (1-structural changes), US (5-education, health coaching, tailored feedback and advice, behaviour change strategies, incentives for healthy behaviours, family history assessment), the Netherlands (3-tailored feedback and action plans, tailored information, tailored motivational interviewing), UK (1-annual health checks), Australia (1-tailored advice), Belgium (1-feedback and advice)  **Other designs:** Turkey (1- education, lifestyle advice), China (1-education), Iran (1- education and structural interventions), Denmark (1-mass communication, group activities), US (1-tailored coaching), the Netherlands (1-mass communication, group activities), UK (1-no intervention details reported) |
| University students  (15 studies) | Health promotion | 9 | **RCTs**: Mexico (1-CBT techniques), US (2-tailored education, education plus goal setting and improvement of self-efficacy), New Zealand (1-assessment, feedback and advice), country not reported (2-education and personalised feedback, coaching, health risk appraisal and personalised feedback)  **Other designs:** US (3-education, peer education, social support, and participation in a health fair) |
|  | Prevention/reduction of risk for chronic disease | 5 | **RCTs**: US (3-self management skills training, tailored education and feedback, CBT skills training, and group role play)  **Other designs:** Jordan (1-education), US (1-education, structural changes) |
|  | Prevention of initiation of risk behaviours | 1 | **Other designs:** US (1- skills and education) |
| Racial and minority ethnic groups  (8 studies) | Health promotion | 3 | **RCTs**: US (2- dancing lessons and dietary education, environmental (i.e., social, cultural, physical) and organizational (i.e., policies, practices) changes within church – included education, bulletins, and policy/practices set by pastors  **Other designs:** US (1-culturally tailored education) |
|  | Weight management | 1 | **Other designs:** US (1- education and behavioural strategies.) |
|  | Prevention/reduction of risk for chronic disease | 3 | **RCTs**: US (3- education and social support, skills training for behaviour change, and counselling) |
|  | Prevention of initiation of risk behaviours | 1 | **RCTs**: US (1-skills training) |
| Older adults  (6 studies ) | Health promotion | 5 | **RCTs**: US (2- health risk appraisals, tailored recommendations and self-management materials, education, self-recording of behaviours and personalised goals), China (1- education, tailored advice and motivational interviewing), Australia (1- lifestyle recommendations, goal setting, various tools, and telephone and email support from program guides.)  **Other designs**: Taiwan (1- education) |
|  | Prevention/reduction of risk for chronic disease | 1 | **RCTs**: US (1- visits to their doctor and counselling) |
| Homeless /low socio-economic status  (6 studies) | Health promotion | 3 | **RCTs**: UK (1-environmental changes)  **Other designs:** Chile (1-dietary education and physical activity sessions), UK (1-motivational interviewing) |
|  | Prevention/reduction of risk for chronic disease | 3 | **RCTs**: US (1- tailored recommendations for behaviour change, counselling, access to local activities, and education tailored for low literacy audiences)  **Other designs:** Australia (1-motivational interviewing, personalised feedback and advice), the Netherlands (1-large umbrella project with 790 interventions including nutrition parties, televised exercise sessions, and education – results reported at the population level) |
| Parents with children  (3 studies) | Health promotion | 3 | **RCTs**: US (1-education, behavioural strategies, telephone support), Australia (1-interactive group education sessions)  **Other designs:** US (1-motivational interviewing, counselling, education and skills training) |
| Pregnant women  (3 studies) | Health promotion | 2 | **RCTs**: Australia (1-education and behaviour change strategies)  **Other designs:** The Netherlands (1-education) |
|  | Weight management | 1 | **RCTs**: Country not reported (1 – exercise sessions and dietary counselling) |
| Young adults other than students (aged 16 to 25 years)  (2 studies) | Prevention/reduction of risk for chronic disease | 1 | **RCTs**: UK (1- motivational interviewing and education) |
|  | Prevention of initiation of risk behaviours | 1 | **RCTs**: UK (1- motivational interviewing |
| Patients from healthcare practices  (2 studies) | Prevention/reduction of risk for chronic disease | 2 | **Other designs:** US (1-this paper summarised findings from 10 intervention studies in different areas, all under the same umbrella project. Most incorporated a form of counselling). China (1-counselling). |
| Women only  (3 studies) | Prevention/reduction of risk for chronic disease | 2 | **RCTs**: US (1- education and guidance, included goal setting, stimulus control, relapse prevention, and cognitive and motivational techniques)  **Other designs:** Israel (1-education, discussion groups and increased access to doctors for personal consultations) |
|  | Health promotion | 1 | **RCTs**: US (1- education and social support from peers via a buddy system) |
| Men only  (1 study) | Prevention/reduction of risk for chronic disease | 1 | **Other designs:** UK (1-education, physical assessment, and advice) |
| Prison inmates  (1 study) | Prevention/reduction of risk for chronic disease | 1 | **RCTs**: US (1- education) |
| Couples undergoing fertility treatment  (1 study) | Health promotion | 1 | **Other designs:** Australia (1-lifestyle assessment and motivational interviewing) |
| Highly educated adults  (1 study) | Prevention/reduction of risk for chronic disease | 1 | **RCTs**: Belgium (1- personalised website and coaching) |
| Armed Forces veterans  (1 study) | Health promotion | 1 | **Other designs:** US (1-interactive, individual sessions that included skills training and education) |
| **TARGETED SUBGROUPS OF THE GENERAL POPULATION (47 STUDIES, WITH SAMPLE SIZES RANGING FROM 20 TO 1,403):GERAL** | | | |
| Overweight/Obese  (33 studies) | Health promotion | 1 | **Other designs:** New Zealand (1-education) |
|  | Weight management | 30 | **RCTs**: US (14-self-monitoring, goal setting, personalised feedback, motivational interviewing, education, self-control skills training, counselling, tailored recommendations, and behaviour therapy), Australia (2-goal setting, self-monitoring and reinforcement, education, tools and personalised feedback), country not reported (1-tailored feedback and counselling), Belgium (1-advice and counselling), the Netherlands (1-web based application, called ‘Healthy Weight Assistant’), UK (1-advice, tools, education and counselling), Japan (2-counselling, exercise sessions, education, self-monitoring and goal setting), Denmark (1-counselling, advice and free membership to fitness centre)  **Other designs:** Australia (1-education and motivational interviewing), UK (1-behaviour change techniques and goal setting), US (2-education, group discussion, record keeping, and computerised dietary assessment), Canada (1-assessment, counselling, tailored diet plan, and a walking program), the Netherlands (1-tailored education)  **Design not reported:** US (1-education and activity materials) |
|  | Prevention/reduction of risk for chronic disease | 2 | **Other designs:** US (2- education, supervised physical activity sessions, goal setting, self-monitoring techniques, skills training) |
| Adult drug users  (11 studies) | Prevention/reduction of risk for chronic disease | 11 | **RCTs**: US (3-education and counselling, personalised feedback, skills training, goal setting, motivational interviewing), country not reported (1-education, behaviour strategies, skills training, support groups, and counselling)  **Other designs:** Russia (1-peer education and HIV-test counselling), US (5-HIV counselling and testing, legal services, and family, recreational and social activities, tailored education, access to resources, and facilitated discussions), China (1-counselling and peer education) |
| University students with (or at risk of) problematic substance use  (2 studies) | Reduction of risk for substance dependence and other problems (e.g., academic impairment) | 2 | **Other designs:** US (2-motivational interviewing, self-monitoring and personalised feedback) |
| Adult smokers with untreated depression (1 study) | Prevention/reduction of risk for chronic disease | 1 | **RCTs**: US (1-CBT-based counselling sessions and activity materials) |
| **AT RISK POPULATION (54 STUDIES, WITH SAMPLE SIZES RANGING FROM 18 TO 13,016):** | | | |
| Cardiovascular disease risk  (11 studies) | Prevention/reduction of risk for chronic disease | 10 | **RCTs**: US (4-education, counselling, tailored newsletters, and goal setting), Denmark (1-personalised lifestyle consultation and counselling)  **Other designs:** US (3-education, counselling, supervised physical activity sessions, self-monitoring, group support, lifestyle recommendations), Japan (1-counselling, education and physical activity sessions), Sweden (1-education, advice, group/club activities, individual consultations) |
|  | Health promotion | 1 | **Other designs:** South Korea (1-education, counselling and group discussions) |
| Diabetes risk  (8 studies) | Prevention/reduction of risk for chronic disease | 8 | **RCTs**: Country not reported (1-no intervention details reported in conference abstract), Finland (1- counselling), US (2-education, behavioural support, motivational interviewing and tailored advice), the Netherlands (1-motivational interviewing)  **Other designs:** Poland (1-education, social support, and motivation sessions), Finland (1-education and counselling), country not reported (1-counselling) |
| Cancer survivors  (5 studies) | Weight management | 1 | **Other designs:** US (1- discussions, group activities and exercise classes.) |
|  | Health promotion | 3 | **RCTs**: Country not reported (1-advice), Australia (1-health coaching)  **Other designs:** Australia (1-health coaching) |
|  | Prevention/reduction of risk for chronic disease | 1 | **Other designs**: US (1-behaviour change classes and counselling) |
| People with hypertension  (5 studies) | Prevention/reduction of risk for chronic disease | 4 | **RCTs**: Australia (1-cognitive behaviour change strategies, goal setting, advice, social support, time management), Italy (1-education), country not reported (1-education), Spain (1-education) |
|  | Health promotion | 1 | **RCTs**: US (1- automated telephone counselling) |
| People with, or at risk of metabolic syndrome  (5 studies) | Prevention/reduction of risk for chronic disease | 4 | **RCTs**: Norway (1-dietary counselling and supervised physical activity sessions), Romania (1- counselling), Italy (1-tailored lifestyle advice)  **Other designs:** France (1-counselling) |
|  | Health promotion | 1 | **RCTs**: Australia (1-education, behavioural strategies, peer group support, skills training, physical activity sessions) |
| Women with, or at risk of gestational diabetes  (3 studies) | Prevention/reduction of risk for chronic disease | 3 | **RCTs**: US (1-goal setting, counselling, self-monitoring, and education), China (1-tailored advice), Australia (1-motivational interviewing) |
| Other (e.g., people who had undergone a colonoscopy)  (13 studies) | Prevention/reduction of risk for chronic disease | 10 | **RCTs**: The Netherlands (1-tailored advice and counselling), UK (1-tailored advice, goal setting and social support), US, UK and Canada (1-tailored advice, personalised record logs, education), Spain (1-education) US (3-counselling, community-based exercise classes, goal setting, support planning with spouses, education, and encouragement to join a smoking cessation program)  **Other designs:** Country not reported (1-counselling), Australia (2-health coaching and use of pedometer, counselling, gym memberships, skills training) |
|  | Weight management | 3 | **RCTs**: The Netherlands (1- counselling, self-monitoring, and personalised feedback), Japan (1-education, goal setting, advice and self-monitoring)  **Other designs:** US (1-advice, self-regulation skills training and social support) |
| A combination of the above  (4 studies) | Prevention/reduction of risk for chronic disease | 4 | **RCTs**: Japan (1-education and goal setting), Sweden (1-supervised physical activity and dietary counselling), US (1-behaviour recommendations), Australia (1-education and goal setting) |
| **WORKSITE STUDIES (36 STUDIES, WITH SAMPLE SIZES RANGING BETWEEN 33 AND 28,000):** | | | |
| All studies with interventions performed in worksites (36 studies)  (Note: These studies did not always provide much detail on the populations involved. Where reported, there was wide variation in the work employees did. Examples included bus driving, office work, teaching, hospital work, and construction). | Health promotion | 18 | **RCTs**: US (6-education, tailored goals, self-monitoring, personal support), country not reported (2-education and payroll-based incentive system, no intervention details reported for the other study), Canada (1-email messages promoting healthy behaviours), the Netherlands (1-tailored lifestyle recommendations)  **Other designs:** Northern Ireland (1-lifestyle assessments), (6-incentives, skills training, motivational interviewing, environmental changes, and education), Canada (1-education) |
|  | Weight management | 8 | **RCTs**: US (2-Counselling, environmental changes), Australia (1- financial incentives and education), the Netherlands (2-, guideline-based care, counselling)  **Other designs:** US (1-lifestyle recommendations, goal setting, problem solving, education, and a walking program), the Netherlands (1-no intervention details reported), South Korea (1-incentives) |
|  | Prevention/reduction of risk for chronic disease | 10 | **RCTs:** US (3-education, skills training, environmental changes, feedback, motivational interviewing), the Netherlands (1-motivational interviewing), Sweden (1-education and counselling)  **Other designs:** Japan (1-counselling, and social and environmental support), country not reported (1-personalised action plan), South Korea (1-education and health behaviour diaries) , US (1-group support, skills training, CBT), Canada (1-goal setting and self-monitoring) |

* RCT=Randomised Controlled Trial, ** CBT=Cognitive Behavioural Therapy,

*** Other (non-RCT) study designs included before and after studies, non-randomised controlled trials, case-control studies, a cohort study, and an interrupted time series.
