# Supplementary material for: Characteristics of Interventions Targeting Multiple Lifestyle Risk Behaviours in Adult Populations: A Systematic Scoping Review
Source: PLoS One. 2015 Jan 24;10(1):e0117015. doi: 10.1371/journal.pone.0117015 (PMC4305300; doi:10.1371/journal.pone.0117015)
Supplement: S3 Table — (DOCX) [file pone.0117015.s005.docx]

**Table S3.** Study characteristics (presented according to study country)

| **STUDY COUNTRY** | **RISK BEHAVIOURS TARGETED** | **TOTAL NUMBER OF STUDIES** | **STUDY POPULATIONS**  **(NUMBER OF STUDIES PER POPULATION)** |
| --- | --- | --- | --- |
| US  (107 studies) | Diet and physical activity | 61 | General adult population [1-7]  University students [8-11]  Racial and minority ethnic groups [12-17]  Older adults [18]  Homeless/low socioeconomic status [19]  Women only [20, 21]  Armed Forces veterans [22]  Overweight/obese [23-39]  Cardiovascular disease risk [40-44]  Diabetes risk [45, 46]  Cancer survivors [47, 48]  People with hypertension [49]  Women with, or at risk of gestational diabetes [50]  Other at risk populations [51, 52]  People with a combination of chronic disease risks [53]  Worksite interventions [54-61] |
|  | Illicit drug use and sexual risk behaviours | 7 | Racial and minority ethnic groups [62]  Adult drug users [63-68] |
|  | Smoking, diet, and physical activity | 6 | General adult population [69, 70]  University students [71]  Cardiovascular disease risk [72]  Other at risk populations [73]  Worksite interventions [74] |
|  | Alcohol use and illicit drug use | 5 | University students [75]  Racial and minority ethnic groups [76]  Adult drug users [77]  University students with (or at risk of) problematic substance use [78, 79] |
|  | Smoking and diet | 5 | Other at risk populations [80]  Worksite interventions [81-84] |
|  | Smoking, alcohol use, diet, and physical activity | 3 | University students [85]  Older adults [86]  Patients from healthcare practices [87] |
|  | Diet, physical activity, and sedentary behaviours | 4 | General adult population [88]  Parents with children [89, 90]  Overweight/obese [91] |
|  | Smoking and physical activity | 3 | Adults smokers with untreated depression [92]  Worksite interventions [93, 94] |
|  | Smoking, alcohol use, diet, physical activity, and seat belt use | 2 | General adult population [95]  Older adults [96] |
|  | Alcohol use, illicit drug use, and sexual risk behaviours | 2 | University students [97]  Adult drug users [98] |
|  | Alcohol use and sexual risk behaviours | 1 | University students [99] |
|  | Alcohol use, diet, illicit drug use, and sexual risk behaviours | 1 | University students [100] |
|  | Smoking, alcohol use, illicit drug use, and sexual risk behaviours | 1 | Prison inmates [101] |
|  | Physical activity and sedentary behaviours | 1 | Overweight/obese [102] |
|  | Smoking, diet, physical activity, and seat belt use | 1 | Worksite interventions [103] |
|  | Smoking, alcohol use, diet, physical activity, and illicit drug use | 1 | Worksite interventions [104] |
|  | Smoking, physical activity, and seat belt use | 1 | Worksite interventions [105] |
|  | Unclear | 2 | Cardiovascular disease risk [106]  Worksite interventions [107] |
| Australia  (18 studies) | Smoking, alcohol use, diet, and physical activity | 3 | General adult population [108]  People with hypertension [109]  Other at risk populations [110] |
|  | Smoking, alcohol use, diet, physical activity and sun protection | 1 | Homeless/low socio-economic status [111] |
|  | Diet and physical activity | 12 | Older adults [112]  Parents with children [113]  Pregnant women [114]  Overweight/obese [115-117]  Cancer survivors [118]  People with, or at risk of metabolic disease [119]  Women with, or at risk of gestational diabetes [120]  Other at risk populations [121]  People with a combination of chronic disease risks [122]  Worksite interventions [123] |
|  | Unclear | 1 | Couples undergoing fertility treatment [124] |
|  | Smoking, alcohol use, diet, physical activity, and sedentary behaviours | 1 | Cancer survivors [125] |
| The Netherlands  (16 studies) | Diet and physical activity | 7 | General adult population [126]  Overweight/obese [127, 128]  Diabetes risk [129]  Other at risk populations [130]  Worksite interventions [131, 132] |
|  | Smoking, diet, and physical activity | 7 | General adult population [133, 134]  Homeless/low socio-economic status [135]  Pregnant women [136]  Other at risk populations [137]  Worksite interventions [138, 139] |
|  | Diet and sedentary behaviours | 1 | Worksite interventions [140] |
|  | Smoking, alcohol use, diet, and sunbathing | 1 | General adult population [141] |
| Country not reported  (14 studies) | Diet, physical activity, and sedentary behaviours | 1 | General adult population [142] |
|  | Diet and physical activity | 8 | University students [143, 144]  Pregnant women [145]  Overweight/obese [146]  Diabetes risk [147, 148]  Cancer survivors [149]  Worksite interventions [150] |
|  | Illicit drug use and sexual risk behaviours | 1 | Adult drug users [151] |
|  | Alcohol use, diet, and physical activity | 1 | People with hypertension [152] |
|  | Smoking, diet, and physical activity | 2 | Other at risk populations [153]  Worksite interventions [154] |
|  | Unclear | 1 | Worksite interventions [155] |
| UK  (11 studies) | Smoking and physical activity | 1 | General adult population [156] |
|  | Smoking, diet and physical activity | 1 | General adult population [157] |
|  | Smoking, alcohol use, diet, and physical activity | 2 | General adult population [158]  Men only [159] |
|  | Diet and physical activity | 5 | Homeless/low socio-economic status [160, 161]  Overweight/obese [162, 163]  Other at risk populations [164] |
|  | Alcohol use and illicit drug use | 1 | Young adults (aged 16-25 years) [165] |
|  | Smoking, alcohol use, and illicit drug use | 1 | Young adults (aged 16-25 years) [166] |
| Japan  (6 studies) | Diet and physical activity | 6 | Overweight/obese [167, 168]  Cardiovascular disease risk [169]  Other at risk populations [170]  People with a combination of chronic disease risks [171]  Worksite interventions [172] |
| China  (5 studies) | Smoking, alcohol use, diet, and physical activity | 2 | General adult population [173]  Patients from healthcare practices [174] |
|  | Smoking and diet | 1 | Older adults [175] |
|  | Illicit drug use and sexual risk behaviours | 1 | Adult drug users [176] |
|  | Diet and physical activity | 1 | Women with, or at risk of gestational diabetes [177] |
| Canada  (5 studies) | Diet and physical activity | 3 | General adult population [178]  Overweight/obese [179]  Worksite interventions [180] |
|  | Smoking, diet, and physical activity | 2 | Worksite interventions [181, 182] |
| Sweden  (4 studies) | Smoking and diet | 1 | General adult population [183] |
|  | Smoking, alcohol use, diet, and physical activity | 1 | Cardiovascular disease risk [184] |
|  | Diet and physical activity | 1 | People with a combination of chronic disease risks [185] |
|  | Unclear | 1 | Worksite interventions [186] |
| New Zealand  (3 studies) | Diet and physical activity | 2 | General adult population [187]  Overweight/obese [188] |
|  | Smoking, alcohol use, diet, and physical activity | 1 | University students [189] |
| Belgium  (3 studies) | Diet and physical activity | 2 | General adult population [190]  Overweight/obese [191] |
|  | Smoking, diet and physical activity | 1 | Highly educated adults [192] |
| Denmark  (3 studies) | Smoking, diet, and physical activity | 1 | General adult population [193] |
|  | Diet and physical activity | 1 | Overweight/obese [194] |
|  | Smoking, alcohol use, diet, and physical activity | 1 | Cardiovascular disease risk [195] |
| South Korea  (3 studies) | Smoking, alcohol use, diet, and physical activity | 2 | Cardiovascular disease risk [196]  Worksite interventions [197] |
|  | Diet and physical activity | 1 | Worksite interventions [198] |
| Finland  (2 studies) | Diet and physical activity | 2 | Diabetes risk [199, 200] |
| Spain  (2 studies) | Diet and physical activity | 1 | People with hypertension [201] |
|  | Smoking, alcohol use, diet, and sunbathing | 1 | Other at risk populations [202] |
| Italy  (2 studies) | Smoking, diet, and physical activity | 1 | People with hypertension [203] |
|  | Diet and physical activity | 1 | People with, or at risk of metabolic syndrome [204] |
| Turkey  (1 study) | Smoking, diet, and sedentary behaviours | 1 | General adult population [205] |
| Iran  (1 study) | Smoking, diet, and physical activity | 1 | General adult population [206] |
| Mauritius  (1 study) | Smoking, alcohol use, diet, and physical activity | 1 | General adult population [207] |
| Vietnam  (1 study) | Smoking, alcohol use, diet, and physical activity | 1 | General adult population [208] |
| Mexico  (1 study) | Diet and physical activity | 1 | University students [209] |
| Jordan  (1 study) | Diet and physical activity | 1 | University students [210] |
| Taiwan  (1 study) | Diet and physical activity | 1 | Older adults [211] |
| Chile  (1 study) | Diet and physical activity | 1 | Homeless/low socio-economic status [212] |
| Israel  (1 study) | Smoking and sunbathing | 1 | Women only [213] |
| Poland  (1 study) | Diet and physical activity | 1 | Diabetes risk [214] |
| Romania  (1 study) | Diet and physical activity | 1 | People with, or at risk of metabolic syndrome [215] |
| Norway  (1 study) | Diet and physical activity | 1 | People with, or at risk of metabolic syndrome [216] |
| France  (1 study) | Diet and physical activity | 1 | People with, or at risk of metabolic syndrome [217] |
| Russia  (1 study) | Illicit drug use and sexual risk behaviours | 1 | Adult drug users [218] |
| Northern Ireland  (1 study) | Smoking, alcohol use, and physical activity | 1 | Worksite interventions [219] |
| US, UK, and Canada  (1 study) | Diet and physical activity | 1 | Other at risk populations [220] |

1. Aldana SG, Greenlaw RL, Diehl HA, Salberg A, Merrill RM, et al. (2006) The behavioral and clinical effects of therapeutic lifestyle change on middle-aged adults. Prev Chronic Dis 3: A05.
2. Kegler MC, Alcantara I, Veluswamy JK, Haardorfer R, Hotz JA, et al. (2012) Results from an intervention to improve rural home food and physical activity environments. Prog 6: 265-277.
3. Keyserling TC, Samuel Hodge CD, Jilcott SB, Johnston LF, Garcia BA, et al. (2008) Randomized trial of a clinic-based, community-supported, lifestyle intervention to improve physical activity and diet: the North Carolina enhanced WISEWOMAN project. Prev Med 46: 499-510.
4. Petrofsky JS, Bonacci J, Bonilla T, Jorritsma R, Morris A, et al. (2004) Effect of a 1-week diet and exercise program on weight and limb girth. J Appl Res 4: 369-379.
5. Prochaska JO, Evers KE, Castle PH, Johnson JL, Prochaska JM, et al. (2012) Enhancing multiple domains of well-being by decreasing multiple health risk behaviors: a randomized clinical trial. Popul Health Manag 15: 276-286.
6. Salamone LM, Cauley JA, Black DM, Simkin-Silverman L, Lang W, et al. (1999) Effect of a lifestyle intervention on bone mineral density in premenopausal women: a randomized trial. Am J Clin Nutr 70: 97-103.
7. Wylie-Rosett J, Swencionis C, Ginsberg M, Cimino C, Wassertheil-Smoller S, et al. (2001) Computerized weight loss intervention optimizes staff time: the clinical and cost results of a controlled clinical trial conducted in a managed care setting. J Am Diet Assoc 101: 1155-1162; quiz 1163-1154.
8. Franko DL, Cousineau TM, Trant M, Green TC, Rancourt D, et al. (2008) Motivation, self-efficacy, physical activity and nutrition in college students: randomized controlled trial of an internet-based education program. Prev Med 47: 369-377.
9. Greene GW, White AA, Hoerr SL, Lohse B, Schembre SM, et al. (2012) Impact of an online healthful eating and physical activity program for college students. Am J Health Promot 27: e47-58.
10. Hager R, George JD, LeCheminant JD, Bailey BW and Vincent WJ (2012) Evaluation of a university general education health and wellness course delivered by lecture or online. Am J Health Promot 26: 263-269.
11. Lee ML (2009) Implementation and evaluation of a health promotion program on university campuses. Diss Abs Int: Section B: The Sciences and Engineering 69: 6703.
12. Backman D, Scruggs V, Atiedu AA, Bowie S, Bye L, et al. (2011) Using a toolbox of tailored educational lessons to improve fruit, vegetable, and physical activity behaviors among African American women in California. J Nutr Educ Behav 43: S75-S85.
13. Campbell MK, James A, Hudson MA, Carr C, Jackson E, et al. (2004) Improving multiple behaviors for colorectal cancer prevention among African American church members. Health Psychol 23: 492-502.
14. Kim KH-c, Linnan L, Campbell MK, Brooks C, Koenig HG, et al. (2008) The WORD (wholeness, oneness, righteousness, deliverance): a faith-based weight-loss program utilizing a community-based participatory research approach. Health Educ Behav 35: 634-650.
15. Lee RE, Mama SK, Medina A, Orlando Edwards R and McNeill L (2011) SALSA : SAving Lives Staying Active to promote physical activity and healthy eating. J Obesity 2011: Article ID 436509.
16. Staten LK, Gregory-Mercado KY, Ranger-Moore J, Will JC, Giuliano AR, et al. (2004) Provider counseling, health education, and community health workers: the Arizona WISEWOMAN project. J Womens Health 13: 547-556.
17. Wilcox S, Parrott A, Baruth M, Laken M, Condrasky M, et al. (2013) The Faith, Activity, and Nutrition Program: a randomized controlled trial in African-American churches. Am J Prev Med 44: 122-131.
18. Wellman NS, Kamp B, Kirk-Sanchez NJ and Johnson PM (2007) Eat better & move more: a community-based program designed to improve diets and increase physical activity among older Americans. Am J Public Health 97: 710-717.
19. Emmons KM, Stoddard AM, Fletcher R, Gutheil C, Suarez EG, et al. (2005) Cancer prevention among working class, multiethnic adults: results of the healthy directions-health centers study. Am J Public Health 95: 1200-1205.
20. Simkin-Silverman LR, Wing RR, Boraz MA, Meilahn EN and Kuller LH (1998) Maintenance of cardiovascular risk factor changes among middle-aged women in a lifestyle intervention trial. Womens Health 4: 255-271.
21. Weisman CS, Hillemeier MM, Symons Downs D, Feinberg ME, Chuang CH, et al. (2011) Improving women’s preconceptional health: long-term effects of the Strong Healthy Women Behavior Change Intervention in the Central Pennsylvania Women’s Health Study. Women's Health Issues 21: 265-271.
22. Whittle J, Hayes A, Eastwood D, Morzinski J, Ertl K, et al. (2010) Effect on health behaviors of a health promotion intervention delivered through veterans service organizations. J Gen Intern Med 25: S264-S265.
23. Alam N (2008) Impact of a community based health information and weight management program on the lifestyle change and prevention of weight gain among African American women. Obesity 16: S152.
24. Befort CA, Donnelly JE, Sullivan DK, Ellerbeck EF and Perri MG (2010) Group versus individual phone-based obesity treatment for rural women. Eat Behav 11: 11-17.
25. Buscemi J, Yurasek AM, Dennhardt AA, Martens MP and Murphy JG (2011) A randomized trial of a brief intervention for obesity in college students. Clinical Obesity 1: 131-140.
26. Carels RA, Darby LA, Cacciapaglia HM and Douglass OM (2004) Reducing cardiovascular risk factors in postmenopausal women through a lifestyle change intervention. J Womens Health 13: 412-426.
27. Fitzgibbon ML, Stolley MR, Schiffer L, Sanchez-Johnsen LAP, Wells AM, et al. (2005) A combined breast health/weight loss intervention for Black women. Prev Med 40: 373-383.
28. Gow RW (2010) Preventing weight gain in first year college students: an Internet-based intervention. Diss Abs Int: Section B: The Sciences and Engineering 70: 7852.
29. Jaber LA, Pinelli NR, Brown MB, Funnell MM, Anderson R, et al. (2011) Feasibility of group lifestyle intervention for diabetes prevention in Arab Americans. Diabetes Res Clin Pract 91: 307-315.
30. LeCheminant JD, Covington NK, Smith J, Lox CL, Kirk EP, et al. (2011) Evaluation of a university-based community outreach weight management program. Popul Health Manag 14: 167-173.
31. Ostbye T, Krause KM, Lovelady CA, Morey MC, Bastian LA, et al. (2009) Active Mothers Postpartum: a randomized controlled weight-loss intervention trial. Am J Prev Med 37: 173-180.
32. Patrick K, Calfas KJ, Norman GJ, Rosenberg D, Zabinski MF, et al. (2011) Outcomes of a 12-month web-based intervention for overweight and obese men. Ann Behav Med 42: 391-401.
33. Pellegrini CA, Verba SD, Otto AD, Helsel DL, Davis KK, et al. (2012) The comparison of a technology-based system and an in-person behavioral weight loss intervention. Obesity 20: 356-363.
34. Rickel KA (2010) Response of African-American and Caucasian women in a rural setting to a lifestyle intervention for obesity. Diss Abs Int: Section B: The Sciences and Engineering 70: 7862.
35. Stolley MR, Fitzgibbon ML, Wells A and Martinovich Z (2004) Addressing multiple breast cancer risk factors in African-American women. J Natl Med Assoc 96: 76-86.
36. Tate DF, Wing RR and Winett RA (2001) Using Internet technology to deliver a behavioral weight loss program. JAMA 285: 1172-1177.
37. Unick JL, Jakicic JM and Marcus BH (2010) Contribution of behavior intervention components to 24-month weight loss. Med Sci Sports Exerc 42: 745-753.
38. Winett RA, Anderson ES, Wojcik JR, Winett SG, Moore S, et al. (2011) Guide to health: A randomized controlled trial of the effects of a completely web-based intervention on physical activity, fruit and vegetable consumption, and body weight. Transl Behav Med 1: 165-174.
39. Yancey AK, McCarthy WJ, Harrison GG, Wong WK, Siegel JM, et al. (2006) Challenges in improving fitness: results of a community-based, randomized, controlled lifestyle change intervention. J Womens Health 15: 412-429.
40. Amundson HA, Butcher MK, Gohdes D, Hall TO, Harwell TS, et al. (2009) Translating the diabetes prevention program into practice in the general community: findings from the Montana Cardiovascular Disease and Diabetes Prevention Program. Diabetes Educ 35: 209-210, 213-204, 216-220.
41. Calderon KS, Smallwood C and Tipton DA (2008) Kennedy Space Center Cardiovascular Disease Risk Reduction Program evaluation. Vasc Health Risk Manag 4: 421-426.
42. Khare MM, Carpenter RA, Huber R, Bates NJ, Cursio JF, et al. (2012) Lifestyle intervention and cardiovascular risk reduction in the Illinois WISEWOMAN Program. J Womens Health 21: 294-301.
43. Marshall DA, Walizer EM and Vernalis MN (2009) Achievement of heart health characteristics through participation in an intensive lifestyle change program (Coronary Artery Disease Reversal Study). J Mol Signal 29: 84-94.
44. Parra-Medina D, Wilcox S, Salinas J, Addy C, Fore E, et al. (2011) Results of the Heart Healthy and Ethnically Relevant Lifestyle trial: a cardiovascular risk reduction intervention for African American women attending community health centers. Am J Public Health 101: 1914-1921.
45. Knowler W, Barrett-Connor E, Fowler S, Hamman R, Lachin J, et al. (2002) Reduction in the incidence of type 2 diabetes with lifestyle intervention or metformin. N Engl J Med 346: 393-403.
46. Whittemore R, Melkus G, Wagner J, Dziura J, Northrup V, et al. (2009) Translating the diabetes prevention program to primary care: a pilot study. Nurs Res 58: 2-12.
47. Stolley MR, Sharp LK, Oh A and Schiffer L (2009) A weight loss intervention for African American breast cancer survivors, 2006. Prev Chronic Dis 6: A22.
48. Young RF, Zora D and Deutsch-Keahey D (2009) Behavioral change in African American breast cancer survivors: results and challenges. Cancer Res 69.
49. Migneault JP, Dedier JJ, Wright JA, Heeren T, Campbell MK, et al. (2012) A culturally adapted telecommunication system to improve physical activity, diet quality, and medication adherence among hypertensive African-Americans: a randomized controlled trial. Ann Behav Med 43: 62-73.
50. Ferrara A, Hedderson MM, Albright CL, Ehrlich SF, Quesenberry CP, Jr., et al. (2011) A pregnancy and postpartum lifestyle intervention in women with gestational diabetes mellitus reduces diabetes risk factors: a feasibility randomized control trial. Diabetes Care 34: 1519-1525.
51. Befort CA, Klemp JR, Austin HL, Perri MG, Schmitz KH, et al. (2012) Outcomes of a weight loss intervention among rural breast cancer survivors. Breast Cancer Res Treat 132: 631-639.
52. Voils CI, Coffman CJ, Yancy WS, Jr., Weinberger M, Jeffreys AS, et al. (2013) A randomized controlled trial to evaluate the effectiveness of CouPLES: a spouse-assisted lifestyle change intervention to improve low-density lipoprotein cholesterol. Prev Med 56: 46-52.
53. Wing RR, Venditti E, Jakicic JM, Polley BA and Lang W (1998) Lifestyle intervention in overweight individuals with a family history of diabetes. Diabetes Care 21: 350-359.
54. French SA, Harnack LJ, Hannan PJ, Mitchell NR, Gerlach AF, et al. (2010) Worksite environment intervention to prevent obesity among metropolitan transit workers. Prev Med 50: 180-185.
55. Hunt MK, Stoddard AM, Barbeau E, Goldman R, Wallace L, et al. (2003) Cancer prevention for working class, multiethnic populations through small businesses: the healthy directions study. Cancer Causes Control 14: 749-760.
56. Kim Y, Pike J, Adams H, Cross D, Doyle C, et al. (2010) Telephone intervention promoting weight-related health behaviors. Prev Med 50: 112-117.
57. LeCheminant JD and Merrill RM (2012) Improved health behaviors persist over two years for employees in a worksite wellness program. Popul Health Manag 15: 261-266.
58. Sorensen G, Barbeau E, Stoddard AM, Hunt MK, Kaphingst K, et al. (2005) Promoting behavior change among working-class, multiethnic workers: results of the healthy directions--small business study. Am J Public Health 95: 1389-1395.
59. Sternfeld B, Block C, Quesenberry CP, Jr., Block TJ, Husson G, et al. (2009) Improving diet and physical activity with ALIVE: a worksite randomized trial. Am J Prev Med 36: 475-483.
60. Thorndike AN, Sonnenberg L, Healey E, Myint-U K, Kvedar JC, et al. (2012) Prevention of weight gain following a worksite nutrition and exercise program: a randomized controlled trial. Am J Prev Med 43: 27-33.
61. Werneburg BL, Herman LL, Preston HR, Rausch SM, Warren BA, et al. (2011) Effectiveness of a multidisciplinary worksite stress reduction programme for women. Stress and Health 27: 356-364.
62. Peragallo N, Gonzalez-Guarda RM, McCabe BE and Cianelli R (2012) The efficacy of an HIV risk reduction intervention for Hispanic women. AIDS Behav 16: 1316-1326.
63. Andersen MD, Hockman EM and Smereck GAD (1996) Effect of a nursing outreach intervention to drug users in Detroit, Michigan. J Drug Issues 26: 619-634.
64. Stein JA, Nyamathi A and Kington R (1997) Change in AIDS risk behaviors among impoverished minority women after a community-based cognitive-behavioral outreach program. J Community Psychol 25: 519-533.
65. Sterk CE, Theall KP, Elifson KW and Kidder D (2003) HIV risk reduction among African-American women who inject drugs: a randomized controlled trial. AIDS Behav 7: 73-86.
66. Stevens SJ, Estrada AL and Estrada BD (1998) HIV sex and drug risk behavior and behavior change in a national sample of injection drug and crack cocaine using women. Women Health 27: 25-48.
67. Wechsberg WM, Lam WKK, Zule WA and Bobashev G (2004) Efficacy of a woman-focused intervention to reduce HIV risk and increase self-sufficiency among African American crack abusers. Am J Public Health 94: 1165-1173.
68. Williams M, McCoy H, Bowen A, Saunders L, Freeman R, et al. (2001) An evaluation of a brief HIV risk reduction intervention using empirically derived drug use and sexual risk indices. AIDS Behav 5: 31-43.
69. Luepker RV, Murray DM, Jacobs DR, Jr., Mittelmark MB, Bracht N, et al. (1994) Community education for cardiovascular disease prevention: risk factor changes in the Minnesota Heart Health Program. Am J Public Health 84: 1383-1393.
70. Ruffin MTt, Nease DE, Jr., Sen A, Pace WD, Wang C, et al. (2011) Effect of preventive messages tailored to family history on health behaviors: the Family Healthware Impact Trial. Ann Fam Med 9: 3-11.
71. Dismuke SE and McClary AM (1990) Evaluation of an educational-program in preventive cardiology. Am J Prev Med 6: 99-105.
72. Hayashi T, Farrell MA, Chaput LA, Rocha DA and Hernandez M (2010) Lifestyle intervention, behavioral changes, and improvement in cardiovascular risk profiles in the California WISEWOMAN project. J Womens Health 19: 1129-1138.
73. Becker DM, Yanek LR, Johnson WR, Jr., Garrett D, Moy TF, et al. (2005) Impact of a community-based multiple risk factor intervention on cardiovascular risk in black families with a history of premature coronary disease. Circulation 111: 1298-1304.
74. Hughes SL, Seymour RB, Campbell RT, Shaw JW, Fabiyi C, et al. (2011) Comparison of two health-promotion programs for older workers. Am J Public Health 101: 883-890.
75. Ametrano IM (1992) An evaluation of the effectiveness of a substance-abuse prevention program. J Coll Stud Dev 33: 507-515.
76. Brody GH, Yu T, Chen YF, Kogan SM and Smith K (2012) The Adults in the Making program: long-term protective stabilizing effects on alcohol use and substance use problems for rural African American emerging adults. J Consult Clin Psychol 80: 17-28.
77. Guydish J, Werdegar D, Sorensen JL, Clark W and Acampora A (1995) A day treatment program in a therapeutic community setting: six-month outcomes. The Walden House Day Treatment Program. J Subst Abuse Treat 12: 441-447.
78. Amaro H, Reed E, Rowe E, Picci J, Mantella P, et al. (2010) Brief screening and intervention for alcohol and drug use in a college student health clinic: feasibility, implementation, and outcomes. J Am Coll Health 58: 357-364.
79. Denering LL and Spear SE (2012) Routine use of screening and brief intervention for college students in a university counseling center. J Psychoactive Drugs 44: 318-324.
80. Williams GC, McGregor H, Sharp D, Kouldes RW, Levesque CS, et al. (2006) A self-determination multiple risk intervention trial to improve smokers' health. J Gen Intern Med 21: 1288-1294.
81. Glasgow RE, Terborg JR, Hollis JF, Severson HH and Boles SM (1995) Take heart: results from the initial phase of a work-site wellness program. Am J Public Health 85: 209-216.
82. Sorensen G, Thompson B, Glanz K, Feng Z, Kinne S, et al. (1996) Work site-based cancer prevention: primary results from the Working Well Trial. Am J Public Health 86: 939-947.
83. Sorensen G, Stoddard A, Hunt MK, Hebert JR, Ockene JK, et al. (1998) The effects of a health promotion-health protection intervention on behavior change: the WellWorks Study. Am J Public Health 88: 1685-1690.
84. Sorensen G, Stoddard AM, LaMontagne AD, Emmons K, Hunt MK, et al. (2002) A comprehensive worksite cancer prevention intervention: behavior change results from a randomized controlled trial (United States). Cancer Causes Control 13: 493-502.
85. Werch CE, Moore MJ, Bian H, DiClemente CC, Huang IC, et al. (2010) Are effects from a brief multiple behavior intervention for college students sustained over time? Prev Med 50: 30-34.
86. Burton LC, Paglia MJ, German PS, Shapiro S and Damiano AM (1995) The effect among older persons of a general preventive visit on three health behaviors: smoking, excessive alcohol drinking, and sedentary lifestyle. Prev Med 24: 492-497.
87. Fernald DH, Dickinson LM, Froshaug DB, Balasubramanian BA, Holtrop JS, et al. (2012) Improving multiple health risk behaviors in primary care: lessons from the Prescription for Health COmmon Measures, Better Outcomes (COMBO) Study. J Am Board Fam Med 25: 701-711.
88. Spring B, Schneider K, McFadden G, Vaughn J, Kozak AT, et al. (2012) Multiple behavior changes in diet and activity a randomized controlled trial using mobile technology. Arch Intern Med 172: 789-796.
89. French SA, Gerlach AF, Mitchell NR, Hannan PJ and Welsh EM (2011) Household obesity prevention: Take Action--a group-randomized trial. Obesity 19: 2082-2088.
90. Taveras EM, Blackburn K, Gillman MW, Haines J, McDonald J, et al. (2011) First steps for mommy and me: a pilot intervention to improve nutrition and physical activity behaviors of postpartum mothers and their infants. Matern Child Health J 15: 1217-1227.
91. Carlson JA, Sallis JF, Ramirez ER, Patrick K and Norman GJ (2012) Physical activity and dietary behavior change in Internet-based weight loss interventions: comparing two multiple-behavior change indices. Prev Med 54: 50-54.
92. McClure JB, Catz SL, Ludman EJ, Richards J, Riggs K, et al. (2011) Feasibility and acceptability of a multiple risk factor intervention: the Step Up randomized pilot trial. BMC Public Health 11: 167.
93. Kanders BS, Ullmann-Joy P, Foreyt JP, Heymsfield SB, Heber D, et al. (1994) The black American lifestyle intervention (BALI): the design of a weight loss program for working-class African-American women. J Am Diet Assoc 94: 310-312.
94. Prochaska JO, Butterworth S, Redding CA, Burden V, Perrin N, et al. (2008) Initial efficacy of MI, TTM tailoring and HRI's with multiple behaviors for employee health promotion. Prev Med 46: 226-231.
95. Kreuter MW and Strecher VJ (1996) Do tailored behavior change messages enhance the effectiveness of health risk appraisal? Results from a randomized trial. Health Educ Res 11: 97-105.
96. Leigh JP, Richardson N, Beck R, Kerr C, Harrington H, et al. (1992) Randomized controlled study of a retiree health promotion program. The Bank of American Study. Arch Intern Med 152: 1201-1206.
97. Sikkema KJ, Winett RA and Lombard DN (1995) Development and evaluation of an HIV-risk reduction program for female college students. AIDS Educ Prev 7: 145-159.
98. Zule WA, Costenbader EC, Coomes CM and Wechsberg WM (2009) Effects of a hepatitis C virus educational intervention or a motivational intervention on alcohol use, injection drug use, and sexual risk behaviors among injection drug users. Am J Public Health 99 Suppl 1: S180-186.
99. Peeler CM (2001) An analysis of the effects of a course designed to reduce the frequency of high-risk sexual behavior and heavy drinking (immune deficiency). Diss Abs Int: Section B: The Sciences and Engineering 61: 5546.
100. White S, Park YS, Israel T and Cordero ED (2009) Longitudinal evaluation of peer health education on a college campus: impact on health behaviors. J Am Coll Health 57: 497-505.
101. Braithwaite RL, Stephens TT, Treadwell HM, Braithwaite K and Conerly R (2005) Short-term impact of an HIV risk reduction intervention for soon-to-be released inmates in Georgia. J Health Care Poor Underserved 16: 130-139.
102. Kennedy BM, Paeratakul S, Champagne CM, Ryan DH, Harsha DW, et al. (2005) A pilot church-based weight loss program for African-American adults using church members as health educators: a comparison of individual and group intervention. Ethn Dis 15: 373-378.
103. Merrill RM, Anderson A and Thygerson SM (2011) Effectiveness of a worksite wellness program on health behaviors and personal health. J Occup Environ Med 53: 1008-1012.
104. Peters KK (1997) Worksite health promotion with campus maintenance workers. Diss Abs Int: Section B: The Sciences and Engineering 58: 2733.
105. Poole K, Kumpfer K and Pett M (2001) The impact of an incentive-based worksite health promotion program on modifiable health risk factors. Am J Health Promot 16: 21-26.
106. Balcazar HG, de Heer H, Rosenthal L, Aguirre M, Flores L, et al. (2010) A Promotores de Salud intervention to reduce cardiovascular disease risk in a high-risk Hispanic border population, 2005-2008. Prev Chronic Dis 7.
107. Holt MC, McCauley M and Paul D (1995) Health impacts of AT&T's Total Life Concept (TLC) program after five years. Am J Health Promot 9: 421-425.
108. Parekh S, Vandelanotte C, King D and Boyle FM (2012) Improving diet, physical activity and other lifestyle behaviours using computer-tailored advice in general practice: a randomised controlled trial. Int J Behav Nutr Phys Act 9: 108.
109. Burke V, Mansour J, Beilin LJ and Mori TA (2008) Long-term follow-up of participants in a health promotion program for treated hypertensives (ADAPT). Nutr Metab Cardiovasc Dis 18: 198-206.
110. Hawkes AL, Patrao TA, Green A and Aitken JF (2012) CanPrevent: a telephone-delivered intervention to reduce multiple behavioural risk factors for colorectal cancer. BMC Cancer 12: 560.
111. Bonevski B, Baker A, Twyman L, Paul C and Bryant J (2012) Addressing smoking and other health risk behaviours using a novel telephone-delivered intervention for homeless people: a proof-of-concept study. Drug Alcohol Rev 31: 709-713.
112. Burke L, Lee A, Jancey J, Xiang L, Kerr D, et al. (2013) Physical activity and nutrition behavioural outcomes of home-based intervention program for seniors: a randomized controlled trial. Int J Behav Nutr Phys Act 10: 14.
113. Lombard CB, Deeks AA, Ball K, Jolley D and Teede HJ (2009) Weight, physical activity and dietary behavior change in young mothers: short term results of the HeLP-her cluster randomized controlled trial. Nutr J 8: 17.
114. Wilkinson SA and McIntyre HD (2012) Evaluation of the 'healthy start to pregnancy' early antenatal health promotion workshop: a randomized controlled trial. BMC Pregnancy Childbirth 12.
115. Aoun S, Osseiran-Moisson R, Shahid S, Howat P and O' Connor M (2012) Telephone lifestyle coaching: is it feasible as a behavioural change intervention for men? J Health Psychol 17: 227-236.
116. Booth AO, Nowson CA and Matters H (2008) Evaluation of an interactive, Internet-based weight loss program: a pilot study. Health Educ Res 23: 371-381.
117. Morgan P, Callister R, Collins C, Plotnikoff R, Young M, et al. (2012) The SHED-IT community trial: a randomised controlled trial of Internet- and paper-based weight loss programs tailored for overweight and obese men. Obes Res Clin Pract 6: 30-31.
118. Hawkes A, Pakenham K, Courneya KS and Patrao T (2011) A randomised controlled trial of the effects of a telephone-delivered program on health behaviours and quality of life for colorectal cancer survivors ('CanChange'). Asia Pac J Clin Oncol 7: 80-81.
119. Pettman TL, Misan GM, Owen K, Warren K, Coates AM, et al. (2008) Self-management for obesity and cardio-metabolic fitness: description and evaluation of the lifestyle modification program of a randomised controlled trial. Int J Behav Nutr Phys Act 5: 53.
120. Reinhardt JA, van der Ploeg HP, Grzegrzulka R and Timperley JG (2012) lmplementing lifestyle change through phone-based motivational interviewing in rural-based women with previous gestational diabetes mellitus. Health Promot J Austr 23: 5-9.
121. Stacey FG, James EL, Chapman K, Lubans DR, Asprey G, et al. (2010) Enrich (exercise and nutrition routine improving cancer health): efficacy and feasibility of an exercise and nutrition program for cancer survivors and carers. Asia Pac J Clin Oncol 6: 145.
122. Kellow N (2011) Evaluation of a rural community pharmacy-based Waist Management Project: bringing the program to the people. Aust J Prim Health 17: 16-22.
123. Morgan PJ, Collins CE, Plotnikoff RC, Cook AT, Berthon B, et al. (2011) Efficacy of a workplace-based weight loss program for overweight male shift workers: the Workplace POWER (Preventing Obesity Without Eating like a Rabbit) randomized controlled trial. Prev Med 52: 317-325.
124. Homan G and Norman R (2010) Implementing a program to assess lifestyle & promote healthy change: the FAST study (Fertility Assessment and Advice Targeting Lifestyle Choices and Behaviours). Aust N Z J Obstet Gynaecol 50: 25-26.
125. Hawkes AL, Gollschewski S, Lynch BM and Chambers S (2009) A telephone-delivered lifestyle intervention for colorectal cancer survivors 'CanChange': a pilot study. Psychooncology 18: 449-455.
126. van Keulen HM, Mesters I, Ausems M, van Breukelen G, Campbell M, et al. (2011) Tailored print communication and telephone motivational interviewing are equally successful in improving multiple lifestyle behaviors in a randomized controlled trial. Ann Behav Med 41: 104-118.
127. Kelders SM, van Gemert-Pijnen JEWC, Werkman A and Seydel ER (2010) Usage and effect of a web-based intervention for the prevention of overweight; a RCT. Stud Health Technol Inform 160: 28-32.
128. van Genugten L, van Empelen P, Boon B, Borsboom G, Visscher T, et al. (2012) Results from an online computer-tailored weight management intervention for overweight adults: randomized controlled trial. J Med Internet Res 14: e44.
129. Vermunt PWA, Milder IEJ, Wielaard F, de Vries JHM, van Oers HAM, et al. (2011) Lifestyle counseling for type 2 diabetes risk reduction in Dutch primary care: results of the APHRODITE study after 0.5 and 1.5 years. Diabetes Care 34: 1919-1925.
130. Driehuis F, Barte JCM, Ter Bogt NCW, Beltman FW, Smit AJ, et al. (2012) Maintenance of lifestyle changes: 3-year results of the Groningen Overweight and Lifestyle study. Patient Educ Couns 88: 249-255.
131. Kwak L, Kremers SPJ, Visscher TLS, van Baak MA and Brug J (2009) Behavioral and cognitive effects of a worksite-based weight gain prevention program: the NHF-NRG in balance-project. J Occup Environ Med 51: 1437-1446.
132. van Wier MF, Ariens GAM, Dekkers JC, Hendriksen IJM, Smid T, et al. (2009) Phone and e-mail counselling are effective for weight management in an overweight working population: a randomized controlled trial. BMC Public Health 9: 6.
133. de Vries H, Kremers SPJ, Smeets T, Brug J and Eijmael K (2008) The effectiveness of tailored feedback and action plans in an intervention addressing multiple health behaviors. Am J Health Promot 22: 417-425.
134. Oenema A, Brug J, Dijkstra A, de Weerdt I and de Vries H (2008) Efficacy and use of an internet-delivered computer-tailored lifestyle intervention, targeting saturated fat intake, physical activity and smoking cessation: a randomized controlled trial. Ann Behav Med 35: 125-135.
135. Wendel-Vos GCW, Dutman AE, Verschuren WMM, Ronckers ET, Ament A, et al. (2009) Lifestyle factors of a five-year community-intervention program: the Hartslag Limburg intervention. Am J Prev Med 37: 50-56.
136. van Dongen JM, van Poppel MNM, Milder IEJ, van Oers HAM and Brug J (2012) Exploring the reach and program use of Hello World, an email-based health promotion program for pregnant women in the Netherlands. BMC Res Notes 5: 514.
137. Broekhuizen K, van Poppel MNM, Koppes LL, Kindt I, Brug J, et al. (2012) Can multiple lifestyle behaviours be improved in people with familial hypercholesterolemia? Results of a parallel randomised controlled trial. PLoS One 7: e50032.
138. Groeneveld IF, Proper KI, van der Beek AJ, Hildebrandt VH and van Mechelen W (2011) Short and long term effects of a lifestyle intervention for construction workers at risk for cardiovascular disease: a randomized controlled trial. BMC Public Health 11: 836.
139. Smeets T, Kremers SPJ, Brug J and de Vries H (2007) Effects of tailored feedback on multiple health behaviors. Ann Behav Med 33: 117-123.
140. Verweij LM, Proper KI, Weel ANH, Hulshof CTJ and van Mechelen W (2012) The application of an occupational health guideline reduces sedentary behaviour and increases fruit intake at work: results from an RCT. Occup Environ Med 69: 500-507.
141. van Assema P, Steenbakkers M, Kok G and Eriksen M (1994) Results of the Dutch community project "Healthy Bergeyk". Prev Med 23: 394-401.
142. Hijazi RR (2012) Exploring the use of smart phones to influence healthy behaviors. Diss Abs Int: Section B: The Sciences and Engineering 72: 5920.
143. Lachausse RG (2012) My student body: effects of an internet-based prevention program to decrease obesity among college students. J Am Coll Health 60: 324-330.
144. Marrone SE (2010) A behavioral health intervention for decreasing weight gain in first year university students. Diss Abs Int: Section B: The Sciences and Engineering 71: 663.
145. Hui A, Back L, Ludwig S, Gardiner P, Sevenhuysen G, et al. (2011) Exercise and dietary intervention increases physical activity, promotes healthy diet and reduces excessive gestational weight gain in pregnant women: a randomized controlled trial in Urban community. Diabetes 60: A351.
146. Gabriele JM (2009) Effects of nondirective and directive support on weight loss in an e-counseling intervention. Diss Abs Int: Section B: The Sciences and Engineering 69: 5777.
147. Kanaya AM, Santoyo-Olsson J, Gregorich S, Grossman M, Moore T, et al. (2012) The Live Well, Be Well study: a community-based, translational lifestyle program to lower diabetes risk factors in ethnic minority and lower-socioeconomic status adults. Am J Public Health 102: 1551-1558.
148. Kulzer B, Hermanns N, Gorges D, Schwarz P and Haak T (2009) Efficacy of a behavioral lifestyle intervention group program (PREDIAS) for the prevention of type 2 diabetes. 69th Annual Meeting of the American Diabetes Association New Orleans. New Orleans, LA United States.
149. Bourke L, Thompson G, Gibson DJ, Daley A, Crank H, et al. (2011) Pragmatic lifestyle intervention in patients recovering from colon cancer: a randomized controlled pilot study. Arch Phys Med Rehabil 92: 749-755.
150. Kantor L, Schomer H and Louw J (1997) Lifestyle changes following a stress management programme: an evaluation. S Afr J Psychol 27: 16-21.
151. McCusker J, Stoddard AM, Hindin RN, Garfield FB and Frost R (1996) Changes in HIV risk behavior following alternative residential programs of drug abuse treatment and AIDS education. Ann Epidemiol 6: 119-125.
152. Iso H, Shimamoto T, Sankai T, Imano H, Koike K, et al. (1994) A randomized controlled trail of intensive and usual community-based education for blood pressure control [Japanese]. Nippon Koshu Eisei Zasshi 41: 1015-1026.
153. Berks D, Hoedjes M, Franx A, Duvekot HJ, Raat H, et al. (2012) Lifestyle intervention after complicated pregnancy successfully improves cardiovascular and metabolic health: results of the pro-active study. Pregnancy Hypertension 2: 192-193.
154. Emmons KM, Linnan LA, Shadel WG, Marcus B and Abrams DB (1999) The Working Healthy Project: a worksite health-promotion trial targeting physical activity, diet, and smoking. J Occup Environ Med 41: 545-555.
155. Jeffery RW, Forster JL, French SA, Kelder SH, Lando HA, et al. (1993) The healthy worker project: a work-site intervention for weight control and smoking cessation. Am J Public Health 83: 395-401.
156. Ussher M, West R, McEwen A, Taylor A and Steptoe A (2003) Efficacy of exercise counselling as an aid for smoking cessation: a randomized controlled trial. Addiction 98: 523-532.
157. Baxter T, Milner P, Wilson K, Leaf M, Nicholl J, et al. (1997) A cost effective, community based heart health promotion project in England: prospective comparative study. BMJ 315: 582-585.
158. Imperial Cancer Research Fund OXCHECK Study Group (1995) Effectiveness of health checks conducted by nurses in primary care: final results of the OXCHECK study. BMJ 310: 1099-1104.
159. Gibbins RL, Riley M and Brimble P (1993) Effectiveness of programme for reducing cardiovascular risk for men in one general practice. BMJ 306: 1652-1656.
160. Hillier FC, Batterham AM, Nixon CA, Crayton AM, Pedley CL, et al. (2012) A community-based health promotion intervention using brief negotiation techniques and a pledge on dietary intake, physical activity levels and weight outcomes: lessons learnt from an exploratory trial. Public Health Nutr 15: 1446-1455.
161. Phillips G, Renton A, Moore DG, Bottomley C, Schmidt E, et al. (2012) The Well London program: a cluster randomized trial of community engagement for improving health behaviors and mental wellbeing: baseline survey results. Trials 13: 105.
162. Dombrowski SU, Sniehotta FF, Johnston M, Broom I, Kulkarni U, et al. (2012) Optimizing acceptability and feasibility of an evidence-based behavioral intervention for obese adults with obesity-related co-morbidities or additional risk factors for co-morbidities: an open-pilot intervention study in secondary care. Patient Educ Couns 87: 108-119.
163. McConnon A, Kirk SF, Cockroft JE, Harvey EL, Greenwood DC, et al. (2007) The Internet for weight control in an obese sample: results of a randomised controlled trial. BMC Health Serv Res 7: 206.
164. Caswell S, Anderson AS and Steele RJC (2009) Bowel health to better health: a minimal contact lifestyle intervention for people at increased risk of colorectal cancer. Br J Nutr 102: 1541-1546.
165. Marsden J, Stillwell G, Barlow H, Boys A, Taylor C, et al. (2006) An evaluation of a brief motivational intervention among young ecstasy and cocaine users: no effect on substance and alcohol use outcomes. Addiction 101: 1014-1026.
166. McCambridge J, Hunt C, Jenkins RJ and Strang J (2011) Cluster randomised trial of the effectiveness of motivational interviewing for universal prevention. Drug Alcohol Depend 114: 177-184.
167. Nakade M, Aiba N, Suda N, Morita A, Miyachi M, et al. (2012) Behavioral change during weight loss program and one-year follow-up: Saku Control Obesity Program (SCOP) in Japan. Asia Pac J Clin Nutr 21: 22-34.
168. Tanaka M, Adachi Y, Adachi K and Sato C (2010) Effects of a non-face-to-face behavioral weight-control program among Japanese overweight males: a randomized controlled trial. Int J Behav Med 17: 17-24.
169. Haruyama Y, Muto T, Nakade M, Kobayashi E, Ishisaki K, et al. (2009) Fifteen-month lifestyle intervention program to improve cardiovascular risk factors in a community population in Japan. Tohoku J Exp Med 217: 259-269.
170. Adachi Y, Sato C, Yamatsu K, Ito S, Adachi K, et al. (2007) A randomized controlled trial on the long-term effects of a 1-month behavioral weight control program assisted by computer tailored advice. Behav Res Ther 45: 459-470.
171. Babazono A, Kame C, Ishihara R, Yamamoto E and Hillman AL (2007) Patient-motivated prevention of lifestyle-related disease in Japan: a randomized, controlled clinical trial. Disease Management & Health Outcomes 15: 119-126.
172. Arao T, Oida Y, Maruyama C, Mutou T, Sawada S, et al. (2007) Impact of lifestyle intervention on physical activity and diet of Japanese workers. Prev Med 45: 146-152.
173. Huang S, Hu X, Chen H, Xie D, Gan X, et al. (2011) The positive effect of an intervention program on the hypertension knowledge and lifestyles of rural residents over the age of 35 years in an area of China. Hypertens Res 34: 503-508.
174. Gongyuan Y, Jianqing P and Shuangfei L (2011) Effect of preventive health intervention program on hypertension from communities in Shenzhen city. Heart 97: A99.
175. Zhou B, Chen K, Yu Y, Wang H, Zhang S, et al. (2010) Individualized health intervention: Behavioral change and quality of life in an older rural Chinese population. Educ Gerontol 36: 919-939.
176. Shen S-Y, Zhang Z-B, Tucker JD, Chang H, Zhang G-R, et al. (2011) Peer-based behavioral health program for drug users in China: a pilot study. BMC Public Health 11: 693.
177. Hu G, Tian H, Zhang F, Liu H, Zhang C, et al. (2012) Tianjin Gestational Diabetes Mellitus Prevention Program: study design, methods, and 1-year interim report on the feasibility of lifestyle intervention program. Diabetes Res Clin Pract 98: 508-517.
178. Culos-Reed SN, Doyle-Baker PK, Paskevich D, Devonish JA and Reimer RA (2007) Evaluation of a community-based weight control program. Physiol Behav 92: 855-860.
179. Mottola MF, Giroux I, Gratton R, Hammond J-A, Hanley A, et al. (2010) Nutrition and exercise prevent excess weight gain in overweight pregnant women. Med Sci Sports Exerc 42: 265-272.
180. Plotnikoff RC, McCargar LJ, Wilson PM and Loucaides CA (2005) Efficacy of an e-mail intervention for the promotion of physical activity and nutrition behavior in the workplace context. Am J Health Promot 19: 422-429.
181. Chung M, Melnyk P, Blue D, Renaud D and Breton M-C (2009) Worksite health promotion: the value of the Tune Up Your Heart program. Popul Health Manag 12: 297-304.
182. Renaud L, Kishchuk N, Juneau M, Nigam A, Tereault K, et al. (2008) Implementation and outcomes of a comprehensive worksite health promotion program. Can J Public Health 99: 73-77.
183. Lingfors H, Lindstrom K, Persson LG, Bengtsson C and Lissner L (2001) Evaluation of "Live for Life", a health promotion programme in the County of Skaraborg, Sweden. J Epidemiol Community Health 55: 277-282.
184. Persson LG, Lindstrom K, Lingfors H and Bengtsson C (1996) Results from an intervention programme dealing with cardiovascular risk factors. Experiences from a study of men aged 33-42 in Habo, Sweden. Scand J Prim Health Care 14: 184-192.
185. Eriksson KM, Westborg C-J and Eliasson MCE (2006) A randomized trial of lifestyle intervention in primary healthcare for the modification of cardiovascular risk factors. Scand J Public Health 34: 453-461.
186. Nilsson PM, Klasson EB and Nyberg P (2001) Life-style intervention at the worksite--reduction of cardiovascular risk factors in a randomized study. Scand J Work Environ Health 27: 57-62.
187. Coppell KJ, Tipene-Leach DC, Pahau HLR, Williams SM, Abel S, et al. (2009) Two-year results from a community-wide diabetes prevention intervention in a high risk indigenous community: the Ngati and Healthy project. Diabetes Res Clin Pract 85: 220-227.
188. Cutler L, King B, McCarthy N, Hamilton G and Cook L (2010) Appetite for life: an evaluation of a primary care lifestyle programme. J Prim Health Care 2: 281-287.
189. Kypri K and McAnally HM (2005) Randomized controlled trial of a web-based primary care intervention for multiple health risk behaviors. Prev Med 41: 761-766.
190. Vandelanotte C, Reeves MM, Brug J and De Bourdeaudhuij I (2008) A randomized trial of sequential and simultaneous multiple behavior change interventions for physical activity and fat intake. Prev Med 46: 232-237.
191. Guelinckx I, Devlieger R, Mullie P and Vansant G (2010) Effect of lifestyle intervention on dietary habits, physical activity, and gestational weight gain in obese pregnant women: a randomized controlled trial. Am J Clin Nutr 91: 373-380.
192. Jacobs N, Clays E, De Bacquer D, De Backer G, Dendale P, et al. (2011) Effect of a tailored behavior change program on a composite lifestyle change score: a randomized controlled trial. Health Educ Res 26: 886-895.
193. Osler M and Jespersen NB (1993) The effect of a community-based cardiovascular-disease prevention project in a Danish municipality. Dan Med Bull 40: 485-485.
194. Vinter CA, Jensen DM, Ovesen P, Beck-Nielsen H and Jorgensen JS (2011) The LiP (Lifestyle in Pregnancy) study: a randomized controlled trial of lifestyle intervention in 360 obese pregnant women. Diabetes Care 34: 2502-2507.
195. Jorgensen T, Borch-Johnsen K, Thomsen TF, Ibsen H, Glumer C, et al. (2003) A randomized non-pharmacological intervention study for prevention of ischaemic heart disease: baseline results Inter99. Eur J Cardiovasc Prev Rehabil 10: 377-386.
196. Ok Ham K and Jeong Kim B (2011) Evaluation of a cardiovascular health promotion programme offered to low-income women in Korea. J Clin Nurs 20: 1245-1254.
197. Hwang GS, Choi JW, Choi SH, Lee SG, Kim KH, et al. (2012) Effects of a tailored health promotion program to reduce cardiovascular disease risk factors among middle-aged and advanced-age bus drivers. Asia Pac J Public Health 24: 117-127.
198. Yoon C, Jung H and Kim Y (2011) Evaluation of an incentive-based obesity management program in a workplace. Int J Occup Saf Ergon 17: 147-154.
199. Absetz P, Valve R, Oldenburg B, Heinonen H, Nissinen A, et al. (2007) Type 2 diabetes prevention in the "real world": one-year results of the GOAL Implementation Trial. Diabetes Care 30: 2465-2470.
200. Lindstrom J, Eriksson JG, Valle TT, Aunola S, Cepaitis Z, et al. (2003) Prevention of diabetes mellitus in subjects with impaired glucose tolerance in the Finnish Diabetes Prevention Study: results from a randomized clinical trial. J Am Soc Nephrol 14: S108-113.
201. Rodríguez MC, Castaño SC, García OL, Recio RJI, Castaño SY, et al. (2009) Efficacy of an educational intervention group on changes in lifestyles in hypertensive patients in primary care: a randomized clinical trial [Spanish]. Rev Esp Salud Publica 83: 441-452.
202. Lopez ML, Iglesias JM, del Valle MO, Comas A, Fernandez JM, et al. (2007) Impact of a primary care intervention on smoking, drinking, diet, weight, sun exposure, and work risk in families with cancer experience. Cancer Causes Control 18: 525-535.
203. Ferrara AL, Pacioni D, Di Fronzo V, Russo BF, Staiano L, et al. (2012) Lifestyle educational program strongly increases compliance to nonpharmacologic intervention in hypertensive patients: a 2-year follow-up study. J Clin Hypertens 14: 767-772.
204. Bo S, Ciccone G, Baldi C, Benini L, Dusio F, et al. (2007) Effectiveness of a lifestyle intervention on metabolic syndrome. A randomized controlled trial. J Gen Intern Med 22: 1695-1703.
205. Arikan I, Metintas S and Kalyoncu C (2011) Application of healthy heart program in the two semi-rural areas in Eskisehir [Turkish]. Anadolu Kardiyoloji Dergisi 11: 485-491.
206. Kelishadi R, Sarrafzadegan N, Sadri GH, Pashmi R, Mohammadifard N, et al. (2011) Short-term results of a community-based program on promoting healthy lifestyle for prevention and control of chronic diseases in a developing country setting: Isfahan Healthy Heart Program. Asia Pac J Public Health 23: 518-533.
207. Dowse GK, Gareeboo H, Alberti KG, Zimmet P, Tuomilehto J, et al. (1995) Changes in population cholesterol concentrations and other cardiovascular risk factor levels after five years of the non-communicable disease intervention programme in Mauritius. Mauritius Non-communicable Disease Study Group. BMJ 311: 1255-1259.
208. Nguyen QN, Pham ST, Nguyen VL, Weinehall L, Wall S, et al. (2012) Effectiveness of community-based comprehensive healthy lifestyle promotion on cardiovascular disease risk factors in a rural Vietnamese population: a quasi-experimental study. BMC Cardiovasc Disord 12: 56.
209. Ulla Diez SM, Fortis AP and Franco SF (2012) Efficacy of a health-promotion intervention for college students: a randomized controlled trial. Nurs Res 61: 121-132.
210. Abu-Moghli FA, Khalaf IA and Barghoti FF (2010) The influence of a health education programme on healthy lifestyles and practices among university students. Int J Nurs Pract 16: 35-42.
211. Hsu H-C, Wang C-H, Chen Y-C, Chang M-C and Wang J (2010) Evaluation of a community-based aging intervention program. Educ Gerontol 36: 547-572.
212. Vio F, Lera L and Zacaria I (2011) Evaluation of a nutrition education and physical activity intervention in Chilean low socioeconomic women [Spanish]. Arch Latinoam Nutr 61: 406-413.
213. Biger C, Epstein LM, Hagoel L, Tamir A and Robinson E (1994) An evaluation of an education programme, for prevention and early diagnosis of malignancy in Israel. Eur J Cancer Prev 3: 305-312.
214. Gilis-Januszewska A, Szybinski Z, Kissimova-Skarbek K, Piwonska-Solska B, Pach D, et al. (2011) Prevention of type 2 diabetes by lifestyle intervention in primary health care setting in Poland: Diabetes in Europe Prevention using Lifestyle, physical Activity and Nutritional intervention (DE-PLAN) project. Br J Diabetes Vasc Dis 11: 198-203.
215. Avram C, Iurciuc M, Craciun L, Avram A, Iurciuc S, et al. (2011) Dietary and physical activity counseling in high-risk asymptomatic patients with metabolic syndrome - A primary care intervention. J Food Agriculture Environ 9: 16-19.
216. Anderssen SA, Carroll S, Urdal P and Holme I (2007) Combined diet and exercise intervention reverses the metabolic syndrome in middle-aged males: results from the Oslo Diet and Exercise Study. Scand J Med Sci Sports 17: 687-695.
217. Bihan H, Takbou K, Cohen R, Michault A, Boitou F, et al. (2009) Impact of short-duration lifestyle intervention in collaboration with general practitioners in patients with the metabolic syndrome. Diabetes Metab 35: 185-191.
218. Broadhead RS, Volkanevsky VL, Rydanova T, Ryabkova M, Borch C, et al. (2006) Peer-driven HIV interventions for drug injectors in Russia: first year impact results of a field experiment. Int J Drug Policy 17: 379-392.
219. Addley K, McQuillan P and Ruddle M (2001) Creating healthy workplaces in Northern Ireland: evaluation of a lifestyle and physical activity assessment programme. Occup Med (Lond) 51: 439-449.
220. Demark-Wahnefried W, Morey MC, Sloane R, Snyder DC, Miller PE, et al. (2012) Reach out to enhance wellness home-based diet-exercise intervention promotes reproducible and sustainable long-term improvements in health behaviors, body weight, and physical functioning in older, overweight/obese cancer survivors. J Clin Oncol 30: 2354-2361.
