# Supplementary material for: Characteristics of Interventions Targeting Multiple Lifestyle Risk Behaviours in Adult Populations: A Systematic Scoping Review
Source: PLoS One. 2015 Jan 24;10(1):e0117015. doi: 10.1371/journal.pone.0117015 (PMC4305300; doi:10.1371/journal.pone.0117015)
Supplement: S4 Table — (DOCX) [file pone.0117015.s006.docx]

**Table S4.** Intervention characteristics (presented according to study country)

| **STUDY POPULATION** | **FOCUS OF THE INTERVENTION** | **STUDY POPULATIONS (NUMBER OF STUDIES PER POPULATION)** | **DESIGNS OF STUDIES (NUMBER OF STUDIES)**  **AND THE TYPE OF INTERVENTION** |
| --- | --- | --- | --- |
| US  (107 studies:  69 RCTs, 37 non-RCT designs, and 1 design not reported) | Health promotion | General adult population (2) | RCTs* (1): Education and peer counselling  Non-RCT designs** (1): Coaching and home visits |
|  | Health promotion | University students (5) | RCTs (2): Tailored education, education plus goal setting, and improvement of self-efficacy  Non-RCT designs (3): Education, peer education, social support, and participation in a health fair |
|  | Health promotion | Racial and minority ethnic groups (3) | RCTs (2): Dancing lessons and dietary education, environmental (i.e., social, cultural, physical) and organizational (i.e., policies, practices) changes within church– included education, bulletins, and policy/practices set by pastors  Non-RCT designs (1): Culturally tailored education |
|  | Health promotion | Older adults (2) | RCTs (2): Health risk appraisals, tailored recommendations, and self-management materials, education, self-recording of behaviours and personalised goals |
|  | Health promotion | Parents with children (2) | RCTs (1): Education, behaviour change strategies, and telephone support  Non-RCT designs (1): Motivational interviewing, counselling, education, and skills training |
|  | Health promotion | Armed Forces veterans (1) | Non-RCT design: Interactive, individual sessions that included skills training and education |
|  | Health promotion | Women only (1) | RCT: Education and social support from peers via a buddy system |
|  | Health promotion | People with hypertension (1) | RCT: Automated telephone counselling |
|  | Health promotion | Worksite interventions (12) | RCTs (6): Education, tailored goals, self-monitoring, and personal support  Non-RCT designs (6): Incentives, skills training, motivational interviewing, environmental changes, and education |
|  | Weight management | General adult population (3) | RCTs (2): Education, advice, CBT***, and goal setting  Non-RCT designs (1): Prescribed diet and exercise activities |
|  | Weight management | Racial and minority ethnic groups (1) | Non-RCT design: Education and behavioural strategies |
|  | Weight management | Overweight/  obese (17) | RCTs (14): Self-monitoring, goal setting, personalised feedback, motivational interviewing, education, self-control skills training, counselling, tailored recommendations, and behaviour therapy  Non-RCT designs (2): Education, group discussion, record keeping, and computerised dietary assessment  Design not reported (1): Education and activity materials |
|  | Weight management | Cancer survivors (1) | Non-RCT design: Discussions, group activities, and exercise classes |
|  | Weight management | Other at risk populations (1) | Non-RCT design: Advice, self-regulation skills training, and social support |
|  | Weight management | Worksite interventions (3) | RCTs (2): Counselling and environmental changes  Non-RCT designs (1): Lifestyle recommendations, goal setting, problem solving, education, and a walking program |
|  | Prevention/reduction of risk for chronic disease | General adult population (6) | RCTs (5): Education, health coaching, tailored feedback and advice, behaviour change strategies, incentives for healthy behaviours, and family history assessment.  Non-RCT designs (1): Tailored coaching |
|  | Prevention/reduction of risk for chronic disease | University students (4) | RCTs (3): Self-management skills training, tailored education and feedback, CBT skills training, and group role play  Non-RCT designs (1): Education and structural changes |
|  | Prevention/reduction of risk for chronic disease | Racial and minority ethnic groups (3) | RCTs (3): Education, social support, skills training for behaviour change, and counselling |
|  | Prevention/reduction of risk for chronic disease | Older adults (1) | RCT: Visits to doctor and counselling |
|  | Prevention/reduction of risk for chronic disease | Homeless/low socio-economic status (1) | RCT: Tailored recommendations for behaviour change, counselling, access to local activities, and education tailored for low literacy audiences |
|  | Prevention/reduction of risk for chronic disease | Patients from  healthcare practices (1) | Non-RCT design: Summary of findings  from 10 intervention studies in different areas, all under the same umbrella project; most included counselling |
|  | Prevention/reduction of risk for chronic disease | Women only (1) | RCT: Education and guidance. Included  goal setting, stimulus control, relapse prevention, and cognitive and motivational techniques |
|  | Prevention/reduction of risk for chronic disease | Prison inmates (1) | RCT: Education |
|  | Prevention/reduction of risk for chronic disease | Overweight/  obese (2) | Non-RCT designs (2): Education,  supervised physical activity sessions, goal setting, self-monitoring techniques, and skills training |
|  | Prevention/reduction of risk for chronic disease | Adult drug users  (8) | RCTs (3): Education, counselling,  personalised feedback, skills training, goal setting, and motivational interviewing  Non-RCT designs (5): HIV counselling and testing, legal services, and family, recreational and social activities |
|  | Prevention/reduction of risk for chronic disease | Adult smokers  with untreated depression (1) | RCT: CBT based counselling sessions and  activity materials |
|  | Prevention/reduction of risk for chronic disease | Cardiovascular  disease risk (7) | RCTs (4): Education, counselling, tailored  newsletters, and goal setting  Non-RCT designs (3): Education, counselling, supervised physical activity sessions, self-monitoring, group support, and lifestyle recommendations |
|  | Prevention/reduction of risk for chronic disease | Diabetes risk (2) | RCTs (2): Education, behavioural support, motivational interviewing, and tailored advice |
|  | Prevention/reduction of risk for chronic disease | Cancer survivors (1) | Non-RCT design: Behaviour change classes and counselling |
|  | Prevention/reduction of risk for chronic disease | Women with, or at risk of gestational diabetes (1) | RCT: Goal setting, counselling, self-monitoring, and education |
|  | Prevention/reduction of risk for chronic disease | Other at risk populations (3) | RCTs (3): Counselling, community-based exercise classes, goal setting, support planning with spouses, education, and encouragement to join a smoking cessation program |
|  | Prevention/reduction of risk for chronic disease | People with a combination of chronic disease risks (1) | RCT: Behaviour recommendations |
|  | Prevention/reduction of risk for chronic disease | Worksite interventions (4) | RCTs (3): Education, skills training, environmental changes, feedback, and motivational interviewing  Non-RCT designs (1): Group support, skills training, and CBT |
|  | Prevention of initiation of risk behaviours | University students (1) | Non-RCT design: Skills and education |
|  |  | Racial and minority ethnic groups (1) | RCT: Skills training |
|  | Reduction of risk for substance dependence and other problems (e.g., academic impairment) | University students with (or at risk of) problematic substance use (2) | Non-RCT designs (2): Motivational interviewing, self-monitoring, and personalised feedback |
| Australia  (18 studies:  12 RCTs and 6 non-RCT designs) | Health promotion | Older adults (1) | RCT: Lifestyle recommendations, goal setting, various tools, and telephone and email support from program guides. |
|  | Health promotion | Parents with children (1) | RCT: Interactive group education sessions |
|  | Health promotion | Pregnant women (1) | RCT: Education and behaviour change strategies |
|  | Health promotion | Couples undergoing fertility treatment (1) | Non-RCT design: Lifestyle assessment and motivational interviewing |
|  | Health promotion | Cancer survivors (2) | RCT (1): Health coaching  Non-RCT designs (1): Health coaching |
|  | Health promotion | People with, or at risk of metabolic disease (1) | RCT: Education, behavioural strategies, peer group support, skills training, and physical activity sessions |
|  | Weight management | Overweight/  obese (3) | RCTs (2): Goal setting, self-monitoring and reinforcement, education, tools, and personalised feedback  Non-RCT designs (1): Education and motivational interviewing |
|  | Weight management | Worksite interventions (1) | RCT: Financial incentives and education |
|  | Prevention/  reduction of risk for chronic disease | General adult population (1) | RCT: Tailored advice |
|  | Prevention/  reduction of risk for chronic disease | Homeless/low socio-economic status (1) | Non-RCT design: Motivational interviewing, personalised feedback and advice |
|  | Prevention/  reduction of risk for chronic disease | People with  hypertension (1) | RCT: Cognitive behaviour change  strategies, goal setting, advice, social support, and time management |
|  | Prevention/  reduction of risk for chronic disease | Women with, or at risk of gestational diabetes (1) | RCT: Motivational interviewing |
|  | Prevention/  reduction of risk for chronic disease | Other at risk populations (2) | Non-RCT designs (2): Health coaching, use of pedometer, counselling, gym memberships, and skills training |
|  | Prevention/  reduction of risk for chronic disease | People with a combination of chronic disease risks (1) | RCT: Education and goal setting |
| The Netherlands  (16 studies: 12 RCTs and 4 non-RCT designs) | Health promotion | Pregnant women (1) | RCT: Education |
|  | Health promotion | Worksite interventions (1) | RCT: Tailored lifestyle recommendations |
|  | Weight management | Overweight/  obese (2) | RCTs (1): Web-based application, called ‘Healthy Weight Assistant’  Non-RCT designs (1): Tailored education |
|  | Weight management | Other at risk populations (1) | RCT: Counselling, self-monitoring, and personalised feedback |
|  | Weight management | Worksite interventions (3) | RCTs (2): Guideline-based care, counselling  Non-RCT designs (1): No intervention details reported |
|  | Prevention/reduction of risk for chronic disease | General adult population (4) | RCTs (3): Tailored feedback, action plans, tailored information, and tailored motivational interviewing  Non-RCT design (1): Mass communication and group activities |
|  | Prevention/reduction of risk for chronic disease | Homeless/low socio-economic status (1) | Non-RCT design: Large umbrella project with 790 interventions, including nutrition parties, televised exercise sessions and education – results reported at the population level |
|  | Prevention/reduction of risk for chronic disease | Diabetes risk (1) | RCT: Motivational interviewing |
|  | Prevention/reduction of risk for chronic disease | Other at risk populations (1) | RCT: Tailored advice and counselling |
|  | Prevention/reduction of risk for chronic disease | Worksite interventions (1) | RCT: Motivational interviewing |
| Country not reported  (14 studies:  10 RCTs and 4 non-RCT designs) | Health promotion | University students (2) | RCTs (2): Education, personalised feedback, coaching, and health risk appraisal |
|  | Health promotion | Cancer survivors (1) | RCT: Advice |
|  | Health promotion | Worksite | RCT (2): Education and payroll-based |
|  |  | interventions (2) | incentive system, no intervention details reported for the other study. |
|  | Weight management | General adult population (1) | Non-RCT design: Smartphone application |
|  | Weight management | Pregnant women (1) | RCT: Exercise sessions and dietary counselling |
|  | Weight management | Overweight/  obese (1) | RCTs (1): Tailored feedback and counselling |
|  | Prevention/reduction of risk for chronic disease | Adult drug users (1) | RCT: Education, behavioural strategies, skills training, support groups, and counselling |
|  | Prevention/reduction of risk for chronic disease | Diabetes risk (2) | RCT (1): No intervention details reported  in conference abstract  Non-RCT design (1): Counselling |
|  | Prevention/reduction of risk for chronic disease | People with hypertension (1) | RCT: Education |
|  | Prevention/reduction of risk for chronic disease | Other at risk populations (1) | Non-RCT design: Counselling |
|  | Prevention/reduction of risk for chronic disease | Worksite interventions (1) | Non-RCT design: Personalised action plan |
| UK  (11 studies: 7 RCTs and 4 non-RCT designs ) | Health promotion | General adult population (1) | RCT: CBT and nicotine replacement therapy |
|  | Health promotion | Homeless/low socio-economic status (2) | RCTs (1): Environmental changes  Non-RCT designs (1): Motivational interviewing |
|  | Weight management | Overweight/  obese (2) | RCTs (1): Advice, tools, education, and counselling  Non-RCT designs (1): Behaviour change techniques and goal setting |
|  | Prevention/reduction of risk for chronic disease | General adult population (2) | RCTs (1): Annual health checks  Non-RCT designs (1): No intervention details reported |
|  | Prevention/reduction of risk for chronic disease | Young adults (aged 16-25 years) (1) | RCT: Motivational interviewing and education |
|  | Prevention/reduction of risk for chronic disease | Men only (1) | Non-RCT design: Education, physical assessment, and advice |
|  | Prevention/reduction of risk for chronic disease | Other at risk populations (1) | RCT: Tailored advice, goal setting, and social support |
|  | Prevention of initiation of risk behaviours | Young adults (aged 16-25 years) (1) | RCT: Motivational interviewing |
| Japan  (6 studies: 4 RCTs and 2 non-RCT designs) | Weight management | Overweight/  obese (2) | RCTs (2): Counselling, exercise sessions, education, self-monitoring, and goal setting |
|  | Weight management | Other at risk populations (1) | RCT: Education, goal setting, advice, and self-monitoring |
|  | Prevention/reduction of risk for chronic disease | Cardiovascular disease risk (1) | Non-RCT design: Counselling, education, and physical activity sessions |
|  | Prevention/reduction of risk for chronic disease | People with a combination of chronic disease risks (1) | RCT: Education and goal setting |
|  | Prevention/reduction of risk for chronic disease | Worksite interventions (1) | Non-RCT design: Counselling, and social and environmental support |
| China  (5 studies: 2 RCTs and 3 non-RCT designs) | Health promotion | Older adults (1) | RCT: Education, tailored advice, and motivational interviewing |
|  | Prevention/reduction of risk for chronic disease | General adult population (1) | Non-RCT design: Education |
|  | Prevention/reduction of risk for chronic disease | Patients from healthcare practices (1) | Non-RCT design: Counselling |
|  | Prevention/reduction of risk for chronic disease | Adult drug users (1) | Non-RCT design: Counselling and peer education |
|  | Prevention/reduction of risk for chronic disease | Women with, or at risk of gestational diabetes (1) | RCT: Tailored advice |
| Canada  (5 studies: 1 RCT and 4 non-RCT designs) | Health promotion | Worksite interventions (2) | RCTs (1): Email messages promoting health behaviours  Non-RCT designs (1): Education |
|  | Weight management | General adult population (1) | Non-RCT design: Education and CBT |
|  | Weight management | Overweight/  obese (1) | Non-RCT design: Assessment, counselling, tailored diet plan, and a walking program |
|  | Prevention/reduction of risk for chronic disease | Worksite interventions (1) | Non-RCT design: Goal setting and self-monitoring |
| Sweden  (4 studies: 2 RCTs and 2 non-RCT designs) | Health promotion | General adult population (1) | Non-RCT design: Education and health examinations |
|  | Prevention/reduction of risk for chronic disease | Cardiovascular disease risk (1) | Non-RCT design: Education, advice, group/club activities, and individual consultations |
|  | Prevention/reduction of risk for chronic disease | People with a combination of chronic disease risks (1) | RCT: Supervised physical activity and dietary counselling |
|  | Prevention/reduction of risk for chronic disease | Worksite interventions (1) | RCT: Education and counselling |
| New Zealand  (3 studies: 1 RCT and 2 non-RCT designs | Health promotion | General adult population (1) | Non-RCT design: Education, community activities, and structural changes |
|  | Health promotion | University students (1) | RCT: Assessment, feedback and advice |
|  | Health promotion | Overweight/  obese (1) | Non-RCT design: Education |
| Belgium  (3 studies: All RCTs) | Weight management | Overweight/obese (1) | RCT: Advice and counselling |
|  | Prevention/reduction of risk for chronic disease | General adult population (1) | RCT: Feedback and advice |
|  | Prevention/reduction of risk for chronic disease | Highly educated adults (1) | RCT: Personalised web site and coaching |
| Denmark  (3 studies: 2 RCTs and 1 non-RCT design) | Weight management | Overweight/  obese (1) | RCT: Counselling, advice, and free membership to fitness centre |
|  | Prevention/reduction of risk for chronic disease | General adult population (1) | Non-RCT design: Mass communication and group activities |
|  | Prevention/reduction of risk for chronic disease | Cardiovascular disease risk (1) | RCT: Personalised lifestyle consultation and counselling |
| South Korea  (3 studies: all non-RCT designs) | Health promotion | Cardiovascular disease risk (1) | Non-RCT design: Education, counselling, and group discussions |
|  | Weight management | Worksite interventions (1) | Non-RCT design: Incentives |
|  | Prevention/reduction of risk for chronic disease | Worksite interventions (1) | Non-RCT design: Education and health behaviour diaries |
| Finland  (2 studies: 1 RCT and 1 non-RCT design) | Prevention/reduction of risk for chronic disease | Diabetes risk (2) | RCTs (1): Counselling  Non-RCT designs (1): Education and counselling |
| Spain  (2 studies: both RCTs) | Prevention/reduction of risk for chronic disease | People with hypertension (1) | RCT: Education |
|  | Prevention/reduction of risk for chronic disease | Other at risk populations (1) | RCT: Education |
| Italy  (2 studies: both RCTs) | Prevention/reduction of risk for chronic disease | People with, or at risk of metabolic disease (1) | RCT: Tailored lifestyle advice |
|  | Prevention/reduction of risk for chronic disease | People with hypertension (1) | RCT: Education |
| Turkey  (1 study with a non-RCT design) | Prevention/reduction of risk for chronic disease | General adult population (1) | Non-RCT design: Education and lifestyle advice |
| Iran  (1 study with a non-RCT design) | Prevention/reduction of risk for chronic disease | General adult population (1) | Non-RCT design: Education and structural interventions |
| Mauritius  (1 RCT) | Prevention/reduction of risk for chronic disease | General adult population (1) | RCT: Structural changes |
| Vietnam  (1 study with a non-RCT design) | Health promotion | General adult population (1) | Non-RCT design: Advertising |
| Mexico  (1 RCT) | Health promotion | University students (1) | RCT: CBT techniques |
| Jordan  (1 RCT) | Prevention/reduction of risk for chronic disease | University students (1) | RCT: Education |
| Taiwan  (1 study with a non-RCT design) | Health promotion | Older adults (1) | Non-RCT design: Education |
| Chile  (1 study with a non-RCT design) | Health promotion | Homeless/low socio-economic status (1) | Non-RCT design: Dietary education and physical activity sessions |
| Israel  (1 study with a non-RCT design) | Prevention/reduction of risk for chronic disease | Women only (1) | Non-RCT design: Education, discussion groups and increased access to doctors for personal consultations |
| Poland  (1 study with a non-RCT design) | Prevention/reduction of risk for chronic disease | Diabetes risk (1) | Non-RCT design: Education , social support, and motivation sessions |
| Romania  (1 RCT) | Prevention/reduction of risk for chronic disease | People with, or at risk of metabolic syndrome (1) | RCT: Counselling |
| Norway  (1 RCT) | Prevention/reduction of risk for chronic disease | People with, or at risk of metabolic syndrome (1) | RCT: Dietary counselling and supervised physical activity sessions |
| France  (1 study with a non-RCT design) | Prevention/reduction of risk for chronic disease | People with, or at risk of metabolic syndrome (1) | Non-RCT design: Counselling |
| Russia  (1 study with a non-RCT design) | Prevention/reduction of risk for chronic disease | Adult drug users (1) | Non-RCT design: Peer education and HIV test counselling |
| Northern Ireland  (1 study with a non-RCT design) | Health promotion | Worksite interventions (1) | Non-RCT design: Lifestyle assessments |
| US, UK and Canada  (1 RCT) | Prevention/reduction of risk for chronic disease | Other at risk populations (1) | RCT: Tailored advice, personalised record logs, and education |

* RCT=Randomised Controlled Trial, **Other (non-RCT) study designs included before and after studies, non-randomised controlled trials, case-control studies, a cohort study, and an interrupted time series.

*** CBT=Cognitive Behavioural Therapy.
